# Nimbolide targets RNF114 to induce the trapping of PARP1 and synthetic lethality in *BRCA*-mutated cancer

**DOI:** 10.1126/sciadv.adg7752

**Published:** 2023-10-25

**Authors:** Peng Li, Yuanli Zhen, Chiho Kim, Zhengshuai Liu, Jianwei Hao, Heping Deng, Hejun Deng, Min Zhou, Xu-Dong Wang, Tian Qin, Yonghao Yu

**Affiliations:** ^1^ Department of Biochemistry, University of Texas Southwestern Medical Center, Dallas, TX 75390, USA.; ^2^ Department of Molecular Pharmacology and Therapeutics, Columbia University Vagelos College of Physicians and Surgeons, New York, NY 10032, USA.

## Abstract

Recent studies have pointed to PARP1 trapping as a key determinant of the anticancer effects of PARP1 inhibitors (PARPi). We identified RNF114, as a PARylation-dependent, E3 ubiquitin ligase involved in DNA damage response. Upon sensing genotoxicity, RNF114 was recruited, in a PAR-dependent manner, to DNA lesions, where it targeted PARP1 for degradation. The blockade of this pathway interfered with the removal of PARP1 from DNA lesions, leading to profound PARP1 trapping. We showed that a natural product, nimbolide, inhibited the E3 ligase activity of RNF114 and thus caused PARP1 trapping. However, unlike conventional PARPi, nimbolide treatment induced the trapping of both PARP1 and PARylation-dependent DNA repair factors. Nimbolide showed synthetic lethality with *BRCA* mutations, and it overcame intrinsic and acquired resistance to PARPi, both in vitro and in vivo. These results point to the exciting possibility of targeting the RNF114-PARP1 pathway for the treatment of homologous recombination-deficient cancers.

## Teaser

Nimbolide inhibits RNF114 to induce PARP1 trapping, and its analogs hold promise as therapeutic agents for *BRCA*^mut^ cancers.

## INTRODUCTION

Cancer cells with homologous recombination (HR) deficiency are known to be exquisitely sensitive to poly[adenosine 5′-diphosphate (ADP)-ribose] polymerase 1 (PARP1) inhibitors (PARPi) (*1*). These synthetic lethality mechanisms have been exploited therapeutically for human malignancies with loss-of-function mutations in HR pathway genes, most notably those with *BRCA1* and *BRCA2* mutations (*1*, *2*). The substantial benefits observed in the clinic have led to a paradigm shift, with four PARPi (olaparib, niraparib, rucaparib, and talazoparib) approved by the Food and Drug Administration (FDA) to treat human cancers with *BRCA1/2* mutations (i.e., breast, ovarian, prostate, and/or pancreatic cancers) (*3*–*6*). Despite the tremendous progresses, results from the recent clinical trials (e.g., the OlympiAD and EMBRACA trials) suggest that the therapeutic response of *BRCA*^mut^ tumors to PARPi could vary, depending on the anatomical origins of the lesions (*7*–*9*). Furthermore, intrinsic and acquired resistance to PARPi are frequently observed in the clinic (*10*, *11*). These data highlight the critical need to better understand the mechanisms underlying the therapeutic efficacy of PARPi to develop improved synthetic lethality-based strategies for a more complete and durable therapeutic response in *BRCA*^mut^ cancers.

Our understanding of the mechanisms of action (MOAs) for PARPi is still evolving. The main enzymatic activity of PARP1 is to catalyze a protein posttranslational modification known as poly(ADP)ribosylation (PARylation) (*12*). PARP1 functions as a critical DNA damage sensor (*13*–*16*). In response to genotoxic stress, PARP1 is recruited to nicked DNA and becomes activated (*1*). The activated PARP1 then catalyzes the PARylation on a large array of substrate proteins (including PARP1 itself). These protein-linked PAR polymers serve as a platform for the recruitment of other signaling molecules (e.g., XRCC1, DNA ligase IIIα/LIG3, and DNA Polβ), thereby seeding the formation of a large protein complex that mediates the repair of DNA single-strand breaks (SSBs) (*17*, *18*).

All FDA-approved PARPi competitively occupy the NAD^+^-binding pocket of PARP1. These compounds, however, simultaneously have two distinct yet interconnected activities (i.e., PARP1 inhibition and PARP1 trapping) (*19*, *20*). By inhibiting the catalytic activity of PARP1, PARPi could kill tumor cells by blocking PARylation-dependent DNA damage response (DDR) signaling (*21*). However, PARPi with similar PARP1 inhibitory activities have markedly different cytotoxicity (*22*, *23*). More recent studies then showed that besides inhibiting PARP1, all FDA-approved PARPi also induce PARP1 trapping (*22*). The recruitment of PARP1 to and its dissociation from DNA lesions are a highly dynamic process (*13*, *24*). Upon the treatment of PARPi, PARP1, however, is retained at the DNA damage site for an extended time (termed “trapping”), resulting in the formation of a trapped PARP1-DNA-PARPi complex (*1*, *22*).

The trapped PARP1 causes the collapse of the replication fork and is known to be highly toxic to cells (*1*). Results from recent genetic and pharmacological experiments showed that the presence of the PARP1 protein with uncompromised DNA-binding activities is required for PARPi-induced DDR, cytotoxicity, and innate immune signaling (*22*, *25*–*28*). These results suggest that PARP1 trapping might function as a key determinant for the anti-tumor effects of PARPi (*20*). However, at the molecular level, PARP1 inhibition and PARP1 trapping are coupled (e.g., via PARP1 auto-PARylation), and the mechanistic nature of PARP1 trapping is incompletely understood.

Here, we performed an unbiased, quantitative mass spectrometry (MS)–based screen to identify protein factors that are relocalized, in a PARylation-dependent manner, to the chromatin during DDR. From this screen, we identified a poorly studied E3 ubiquitin ligase, RNF114, that showed PARylation-dependent recruitment to DNA lesions. Using a series of biochemical assays, we identified PARP1 as a previously unknown substrate of RNF114, and RNF114 targets PARylated-PARP1 for ubiquitination and proteasomal degradation. The genetic deletion of RNF114 leads to potent PARP1 trapping, suggesting that blockade of this pathway could be a key contributing factor of PARP1 trapping. Nimbolide, a natural product derived from the Neem tree (*Azadirachta indica*), targets the E3 ubiquitin ligase activity of RNF114 and thereby induces the trapping of PARP1. Although regular nicotinamide adenine dinucleotide (NAD^+^)–competitive PARPi induce the trapping of PARP1, nimbolide treatment causes the trapping of PARP and also PAR-dependent DNA repair factors (i.e., XRCC1). We showed that nimbolide treatment is synthetically lethal in *BRCA*-deficient tumor cells. Nimbolide is also able to kill *BRCA*-deficient tumor cells with intrinsic and acquired resistance to conventional PARPi. This unique MOA points to the exciting possibility of translating nimbolide and its analogs into potential therapeutic agents against *BRCA*-deficient cancers.

## RESULTS

### Comprehensive identification of the PARylation-dependent DNA repair factors

**Fig. 1.**
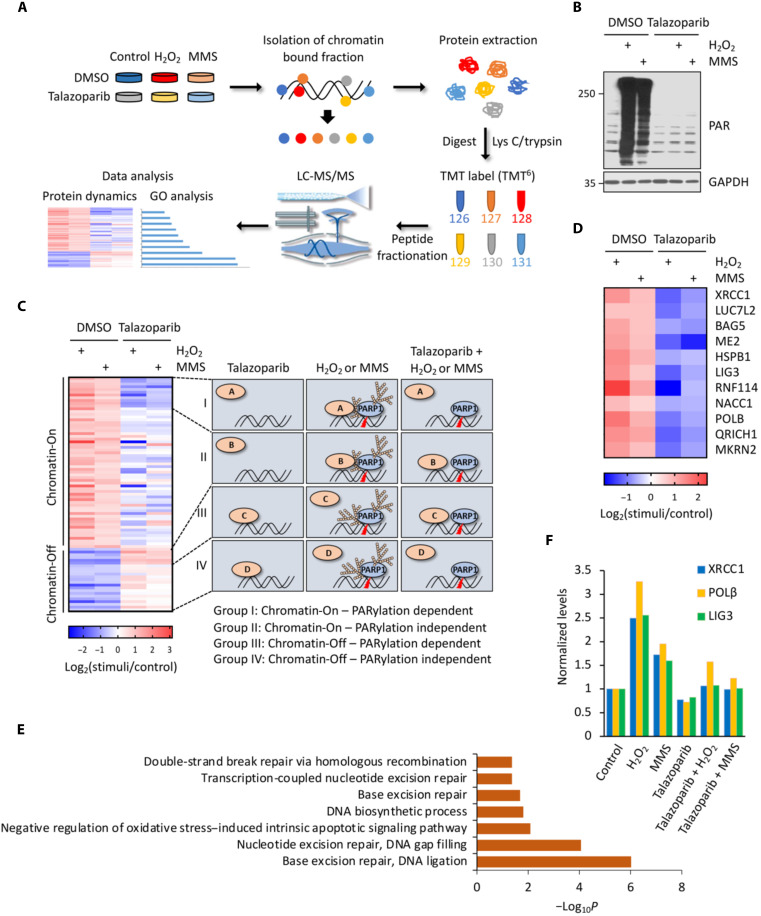
A chromatin relocalization screen for the identification of PARylation-dependent DNA repair factors. (**A**) Overall scheme of the experimental procedures for chromatin relocalization screen. (**B**) Immunoblot analyses of the whole-cell lysate samples derived from (A). (**C**) Hierarchical clustering of the relocalized proteins in response to genotoxic stress. The identified proteins were further classified into four categories, i.e., group I (Chromatin-On; PARylation dependent); group II (Chromatin-On; PARylation independent); group III (Chromatin-Off; PARylation dependent); and group IV (Chromatin-Off; PARylation independent). See the main text for the detailed description of these four classes of proteins. (**D**) A detailed heatmap of all group I proteins. (**E**) Gene Ontology (GO) analyses of the group I proteins (Chromatin-On; PARylation-dependent proteins) identified from the chromatin relocalization screen. (**F**) Abundances of representative group I proteins (i.e., XRCC1, POLβ, and LIG3) in the chromatin fraction as determined from the chromatin relocalization screen. LC-MS/MS, liquid chromatography tandem mass spectrometry; GAPDH, glyceraldehyde-3-phosphate dehydrogenase;MMS, methyl methanesulfonate; DMSO, dimethyl sulfoxide; TMT, tandem mass tag.

We performed a chromatin relocation screen with the goal of identifying the factors involved in the PARylation-dependent DNA damage response (Fig. 1A). In this case, we used quantitative mass spectrometric profiling experiments to comprehensively characterize the chromatin-associated proteome in cells treated with genotoxic agents that activate PARP1. We pretreated the HCT116 cells with dimethyl sulfoxide (DMSO) or talazoparib (a potent PARPi) (*29*). The cells were then treated with H_2_O_2_ or methyl methanesulfonate (MMS) to induce DNA damage and the subsequent PARP1 activation. Immunoblot analyses showed that the treatment of H_2_O_2_ or MMS induced potent PARP1 activation, which, however, was blocked by talazoparib pretreatment (Fig. 1B). For the quantitative proteomic profiling experiments, we harvested the treated cells and isolated the chromatin fraction. After protein extraction and proteolytic digestion, we used isobaric labeling-based quantitative MS for global protein expression profiling experiments (*30*). Specifically, the digested peptides were labeled with the corresponding tandem mass tag (TMT) reagent. These samples were combined, which were then subject to multidimensional high-performance liquid chromatography (HPLC) separation coupled with quantitative mass spectrometric experiments (*31*). From the combined dataset for the quantitative proteomic MS experiments, we were able to identify and quantify a total of 2346 proteins from these chromatin fractions (table S1).

The identified proteins were subject to unsupervised hierarchical clustering analyses (Fig. 1C and fig. S1). The results showed that these proteins converged into four clusters: Group I: Chromatin-On, PARylation-dependent proteins, i.e., those with increased abundances [log_2_FC > 1; fold change (FC)] in the chromatin samples isolated from the MMS/H_2_O_2_-treated cells compared to control cells. The increase, however, was abolished in the cells that were pretreated with talazoparib; group II: Chromatin-On, PARylation-independent proteins, i.e., those with increased abundances (log_2_FC > 1) in the chromatin samples isolated from the MMS/H_2_O_2_-treated cells compared to control cells. The increase, however, was not affected by talazoparib pretreatment; group III: Chromatin-Off, PARylation-dependent proteins, i.e., those with decreased abundances (log_2_FC < −1) in the chromatin samples isolated from MMS/H_2_O_2_-treated cells compared to control cells. The decrease, however, was abolished in the cells that were pretreated with talazoparib; and group IV: Chromatin-Off, PARylation-independent proteins, i.e., those with decreased abundances (log_2_FC < −1) in the chromatin samples isolated from MMS/H_2_O_2_-treated cells compared to control cells. The decrease, however, was not affected by talazoparib pretreatment.

We performed biochemical experiments to validate the results from our quantitative proteomic screen. We selected several representative hits from our screen (e.g., INTS5, INTS3, SOX11, ZC3H3, and TAF10), and the results showed that an excellent agreement was achieved between the proteomic and biochemical experiments (fig. S2). In summary, our chromatin proteomic screen serves as an invaluable approach for the identification of potential PARylation-dependent and -independent DNA repair factors.

### Identification of RNF114 as a PAR-binding E3 ubiquitin ligase

Among the four clusters, we were focused on the group I proteins (i.e., the Chromatin-On, PARylation-dependent proteins) (Fig. 1D). We performed Gene Ontology analyses of this group of proteins (Fig. 1E and fig. S1) and found that most of these proteins were nuclear proteins involved in DDR-related biological processes, including base excision repair and DNA ligation (*P* = 9.57 × 10^−7^) as well as nucleotide excision repair and DNA gap filling (*P* = 8.75 × 10^−5^) (Fig. 1E). This group contained several DNA repair factors (e.g., XRCC1, POLβ, and LIG3) that are known to be recruited in a PARylation-dependent manner to chromatin in response to DNA damage (Fig. 1, D and F). The identification of these known PARylation-dependent DNA repair factors demonstrated the validity of our screen.

**Fig. 2.**
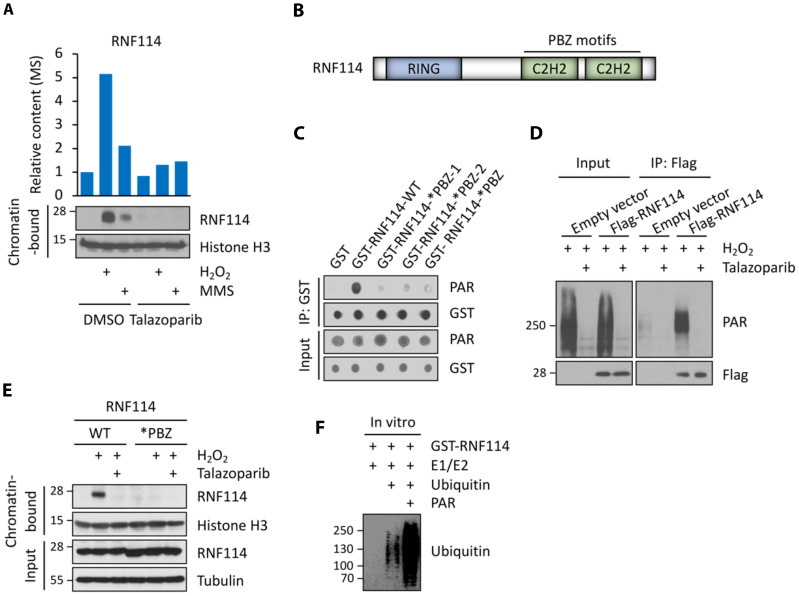
Identification of RNF114 as a PAR-binding, E3 ubiquitin ligase. (**A**) Abundances of RNF114 in the chromatin fraction as determined from the chromatin relocalization screen (top) and biochemical assays (bottom). (**B**) Domain structure of RNF114. (**C** and **D**) RNF114 binds to PAR polymers in vitro (C) and in cells (D). (C) Both RNF114-WT and the various RNF114 mutants were analyzed using the PAR dot blot assay (i.e., RNF114-WT; RNF114-*PBZ-1, C143A/C146A; RNF114-*PBZ-2, C173A/C176A; RNF114-*PBZ, C143A/C146A/C173A/C176A). (D) HCT116 cells expressing the empty vector or Flag-RNF114 were pretreated with talazoparib (1 μM for 1 hour) and then were treated with H_2_O_2_ (2 mM for 5 min). Cell lysates were subject to immunoprecipitation (IP) using an anti-FLAG antibody, and the immunoprecipitants were analyzed using the indicated antibodies. (**E**) PAR binding mediates the translocation of RNF114 to chromatin in response to genotoxic stress. RNF114-KO HCT116 cells were reconstituted with either RNF114-WT or RNF114-*PBZ mutant (the PAR-binding mutant). These cells were pretreated with talazoparib (1 μM for 1 hour) and then were treated with H_2_O_2_ (2 mM for 5 min). The chromatin-bound fraction was isolated from these cells and was subjected to immunoblotting experiments using the indicated antibodies. (**F**) PAR polymers stimulate the E3 activity of RNF114. Recombinant RNF114 was subject to in vitro ubiquitination experiments in the presence (0.4 μM) or absence of added PAR polymers. Immunoblot experiments were used to analyze RNF114 auto-ubiquitination.

Among the other group I proteins, we were particularly intrigued by a protein called RNF114. RNF114 (also known as ZNF313) is a poorly studied E3 ubiquitin-protein ligase with largely unknown functions (*32*, *33*). A previous study showed that RNF114 interacts with A20 and modulates the nuclear factor κB pathway and T cell activation (*34*). However, the role of RNF114 in DDR has not been defined. We performed independent biochemical studies and showed that RNF114 was indeed recruited to the chromatin during DDR (Fig. 2A). Its recruitment, however, was blocked in cells that were pretreated with PARPi. Our proteomic and biochemical studies, therefore, suggest that RNF114 could be a potential factor involved in PARylation-dependent DNA damage response.

RNF114 has several distinct protein domains, including an N-terminal RING domain (an E3 ligase domain), which is followed by two C2H2 [Cys(2)-His(2)]–type zinc finger domains (Fig. 2B). We then used a dot blot assay to biochemically test whether RNF114 is a PAR-binding protein. We found that only RNF114 wild type (WT), but not the RNF114 PAR-binding zinc finger (PBZ) mutants (*PBZ-1, C143A/C146A; *PBZ-2, C173A/C176A; *PBZ, C143A/C146A/C173A/C176A), interacted with PAR polymers (Fig. 2C). Furthermore, we treated cells with H_2_O_2_ to induce DNA damage and to initiate PARylation. Consistent with the notion that RNF114 is a potential PAR-binding protein, a strong PAR signal was detected in the RNF114 immunoprecipitants (Fig. 2D). Furthermore, we performed biochemical fractionation experiments and found that either pretreatment of talazoparib or PBZ mutation completely abolished H_2_O_2_-induced chromatin translocation of RNF114 (Fig. 2E). Together, these findings suggest that the recruitment of RNF114 to the DNA lesions is dependent on its PAR-binding domains.

Previous studies reported that RNF114 is an E3 ligase that is able to modify itself and certain substrate proteins (e.g., A20 and p21) by ubiquitination (*32*, *35*, *36*). Consistent with these studies, we found that RNF114 was able to catalyze its auto-ubiquitination in an in vitro ubiquitination assay (Fig. 2F). Because RNF114 interacts with PAR chains, we further tested whether PAR is a regulator of the E3 ligase activity of RNF114. We found that PAR chains markedly stimulated the E3 ligase activity of RNF114 (Fig. 2F). These results suggest that PAR binding could be involved in regulating both the recruitment and activation of RNF114.

### RNF114 targets PARylated-PARP1 for ubiquitin-proteasomal degradation

**Fig. 3.**
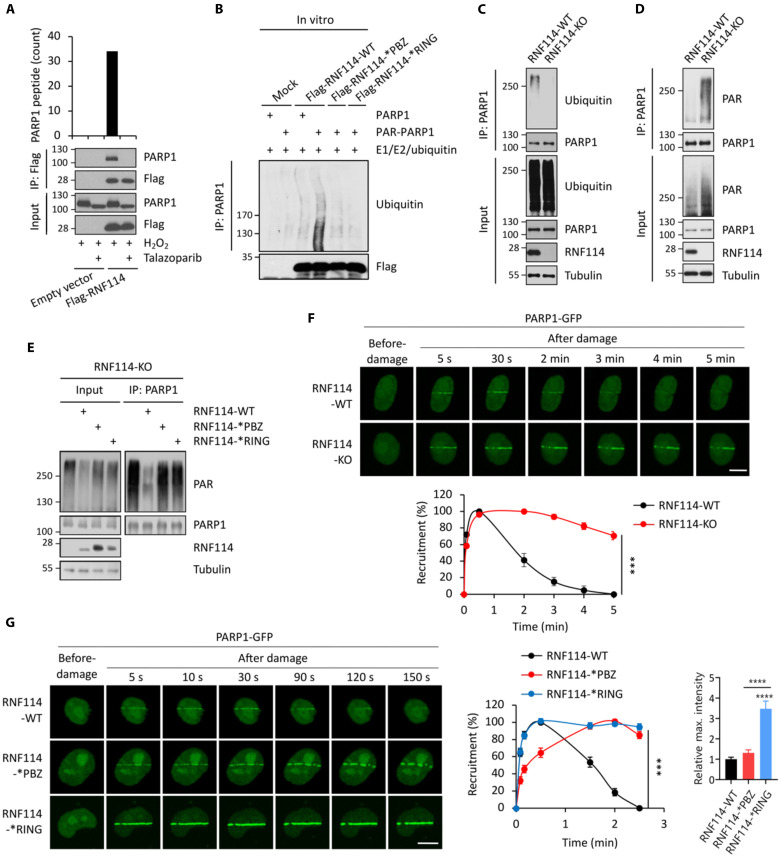
RNF114 targets PARylated-PARP1 for ubiquitin-proteasomal degradation. (**A**) RNF114 interacts with PARylated-PARP1. HCT116 cells expressing the empty vector or Flag-RNF114 were pretreated with talazoparib (1 μM for 1 hour) and were treated with H_2_O_2_ (2 mM for 5 min). The whole-cell lysates were subjected to immunoprecipitation (IP) (anti-Flag), and the immunoprecipitants were analyzed by liquid chromatography tandem mass spectrometry (LC-MS/MS) experiments (top) and immunoblot experiments (bottom). (**B**) In vitro ubiquitination assays of PARP1 or PARylated-PARP1. Purified RNF114-WT, RNF114-*PBZ mutant, or RNF114-*RING mutant was subjected to in vitro ubiquitination experiments in the presence of PARylated-PARP1. (**C**) RNF114 mediates the ubiquitination of PARP1. RNF114-WT and RNF114-KO HCT116 cells were pretreated with MG132 (10 μM for 6 hours) and then were treated with H_2_O_2_ (2 mM for 5 min). PARP1 was isolated using IP and was subject to immunoblotting analyses. (**D**) RNF114 mediates the degradation of PARylated-PARP1. RNF114-WT and RNF114-KO HCT116 cells were treated with H_2_O_2_ (2 mM for 5 min). PARP1 was isolated using IP, and was subject to immunoblotting analyses. (**E**) RNF114 with the uncompromised PAR-binding and E3 ligase activity is required for the degradation of PARylated-PARP1. RNF114-KO HCT116 cells were reconstituted with RNF114-WT, RNF114-*PBZ mutant, or RNF114-*RING mutant. These cells were pretreated with H_2_O_2_ (2 mM for 5 min). PARP1 was isolated using IP and was subjected to immunoblotting analyses. (**F**) Deletion of RNF114 leads to PARP1 trapping. RNF114-WT and RNF114-KO HeLa cells expressing green fluorescent protein (GFP)–tagged PARP1 were subjected to the laser microirradiation experiment. (**G**) Interference with the PAR-binding or the E3 activity of RNF114 leads to PARP1 trapping. RNF114-KO HeLa cells were reconstituted with the RNF114-WT, RNF114-*PBZ mutant, or RNF114-*RING mutant. These cells were transfected with GFP-tagged PARP1 and were subjected to a laser microirradiation assay. GFP signals were monitored and were quantified in a time-course experiment. Scale bars, 10 μm. KO, knockout.

Although RNF114 is known to ubiquitinate several proteins (e.g., A20 and p21) (*32*, *34*), its substrate profile is poorly defined. In particular, it is unclear whether RNF114 targets certain proteins for ubiquitination and degradation to mediate its potential functions in DDR. To identify the substrates of RNF114 in the context of PARylation-mediated DDR, we performed an IP-MS (immunoprecipitation coupled with MS) experiment using cells treated with either H_2_O_2_ or H_2_O_2_ + talazoparib. We identified a large number of PARP1 peptides from the RNF114 immunoprecipitants only in H_2_O_2_-treated cells (Fig. 3A). These results were subsequently validated using immunoblotting experiments (Fig. 3A). However, the interaction between RNF114 and PARP1 was completely blocked by talazoparib pretreatment (Fig. 3A). Consistent with the notion that RNF114 is a PAR-binding protein (Fig. 2C) and that a major fraction of cellular PAR chains are attached to PARP1 itself, our results suggest that PARylated-PARP1, but not PARP1, could directly be involved in binding to RNF114 and may therefore mediate its recruitment to DNA lesions. However, our results do not exclude the possibility that other PARylated proteins could also be involved in regulating the recruitment of RNF114.

Next, we tested the hypothesis that RNF114 could target PARylated-PARP1 for ubiquitin and its subsequent proteasomal degradation. We performed in vitro ubiquitination experiments and found that RNF114 ubiquitinated the PARylated-PARP1 (PAR-PARP1) (Fig. 3B). In addition, we also found that either PBZ or RING mutation completely abolished the RNF114-mediated ubiquitination of PARylated-PARP1 (Fig. 3B), suggesting that both PAR binding motifs and E3 ligase motifs are essential for the ubiquitination of PARylated-PARP1 by RNF114 (Fig. 3B). Moreover, carboxyl-terminus of Hsc70-interacting protein (CHIP) is an E3 ubiquitin ligase that targets misfolded chaperone substrates toward proteasomal degradation (*37*, *38*), and CHIP does not have a PAR binding motif. By using in vitro ubiquitination assays, we found that PAR-PARP1 markedly stimulated the E3 ligase activity of RNF114, but not CHIP, as a negative control (fig. S3A). Therefore, these results indicate that PARylation plays a critical role in regulating both the recruitment and activation and RNF114.

To further investigate this in intact cells, we pretreated control or RNF114–knockout (KO) cells with MG132 to block protein degradation. We then stimulated these cells with H_2_O_2_ to activate PARP1 and isolated PARP1 using IP. We found that the ubiquitination signal in PARP1 immunoprecipitants was markedly decreased in RNF114-KO cells compared to that in the control cells (Fig. 3C). These results suggest that RNF114 is a major E3 ligase that mediates the ubiquitination of PARP1 under genotoxic conditions. In another experiment, we treated control or RNF114-KO cells with H_2_O_2_ to induce DNA damage and the activation of PARP1. Consistent with the potential role of RNF114 in mediating the degradation of PARylated-PARP1, we found that the PAR signal in PARP1 immunoprecipitants was markedly decreased in control cells compared to that in the RNF114-KO cells (Fig. 3D).

Because the PBZ domain is a PAR-binding motif (Fig. 2C) (*15*), we first confirmed that PARylated-PARP1 was bound strongly to RNF114-WT or the RNF114-*RING mutant, but not the RNF114-*PBZ mutant (fig. S3B). Next, we reconstituted the RNF114-KO cells with RNF114-WT, RNF114-*RING (the RING mutant, C29A/C32A, compromised in its E3 ligase activity) (*39*), or RNF114-*PBZ (the PBZ mutant, C143A/C146A/C173A/C176A, compromised in its PAR-binding ability). PARylated-PARP1 was only degraded in cells expressing RNF114-WT, but not those expressing the RNF114-*RING mutant, or the RNF114-*PBZ mutant (Fig. 3E). The degradation of PARylated-PARP1 was completely blocked by MG132 pretreatment, suggesting that PARylated-PARP1 was degraded via the ubiquitin-proteasomal system (fig. S3C). Together, these results demonstrate that RNF114 is a PAR-dependent E3 ligase that targets PARylated-PARP1 for degradation via the ubiquitin proteasome pathway.

### RNF114 blockade results in PARP1 trapping

Using laser microirradiation assays, we found that RNF114 colocalized with proliferating cell nuclear antigen (PCNA) upon microirradiation, further suggesting that RNF114 is involved in the DNA damage repair process (fig. S3D). Next, we performed laser microirradiation assays to examine how RNF114 regulates the recruitment and retention of PARP1 during DDR. In control cells, PARP1 was recruited to DNA lesions within a few seconds and then the PARP1 signal disappeared from DNA lesions after ~5 min (Fig. 3F). However, PARP1 remained at the DNA lesions for a prolonged time in RNF114-KO cells (Fig. 3F), which is consistent with the formation of trapped PARP1. These data are also consistent with a model where RNF114 is recruited, in a PARylation-dependent manner, to the DNA damage site, where it removes PARP1 via the ubiquitin-proteasomal mechanism. The blockage of this pathway instead causes PARP1 trapping.

In addition, we also examined the kinetics of PARP1 relocation in the RNF114-KO cells reconstituted with RNF114-WT, the RNF114-*PBZ mutant, or the RNF114-*RING mutant. Compared to RNF114-WT cells, PARP1 in RNF114-*PBZ mutant cells was retained on DNA lesions for a prolonged time (i.e., PARP1 trapping). This is likely due to the PAR binding deficiency and hence the compromised PAR-mediated recruitment of this RNF114 mutant (Fig. 3G). PARP1 was also retained at the DNA lesions for a prolonged time in cells expressing the RNF1114-*RING mutant. This is likely due to its compromised E3 ligase activity (Fig. 3G). The RNF114-*RING mutant induced a stronger level of PARP1 trapping compared to the RNF114-*PBZ mutant (Fig. 3G). These data are consistent with a potential dominant-negative effect of the RNF114-*RING mutant on PARP1 trapping. Specifically, it is likely that although this mutant does not degrade PARylated-PARP1, it occupies PARylated-PARP1 through the intact PBZ motifs. This binding could protect PARylated-PARP1 and prevent it from being removed from DNA lesions by other PAR-dependent mechanisms.

PARP1 trapping causes the stalling and collapse of replication forks and is known to a key mechanism driving the cytotoxicity of PARPi (*22*). Consistent with the role of RNF114 in regulating PARP1 trapping, we found that compared to control cells, RNF114-KO cells were more susceptible to various genotoxic agents (e.g., H_2_O_2_) (fig. S3E). Furthermore, MMS-induced cell death was ameliorated in RNF114-KO cells reconstituted with RNF114-WT, but not the RNF114-*RING mutant or the RNF114-*PBZ mutant (fig. S3F). Consistent with the ability of RNF114-*RING to induce potent PARP1 trapping, cells expressing the RNF114-*RING mutant showed the most profound levels of cell death under genotoxic conditions (fig. S3F).

### Nimbolide traps PARP1 and the PAR-dependent DNA repair factors

**Fig. 4.**
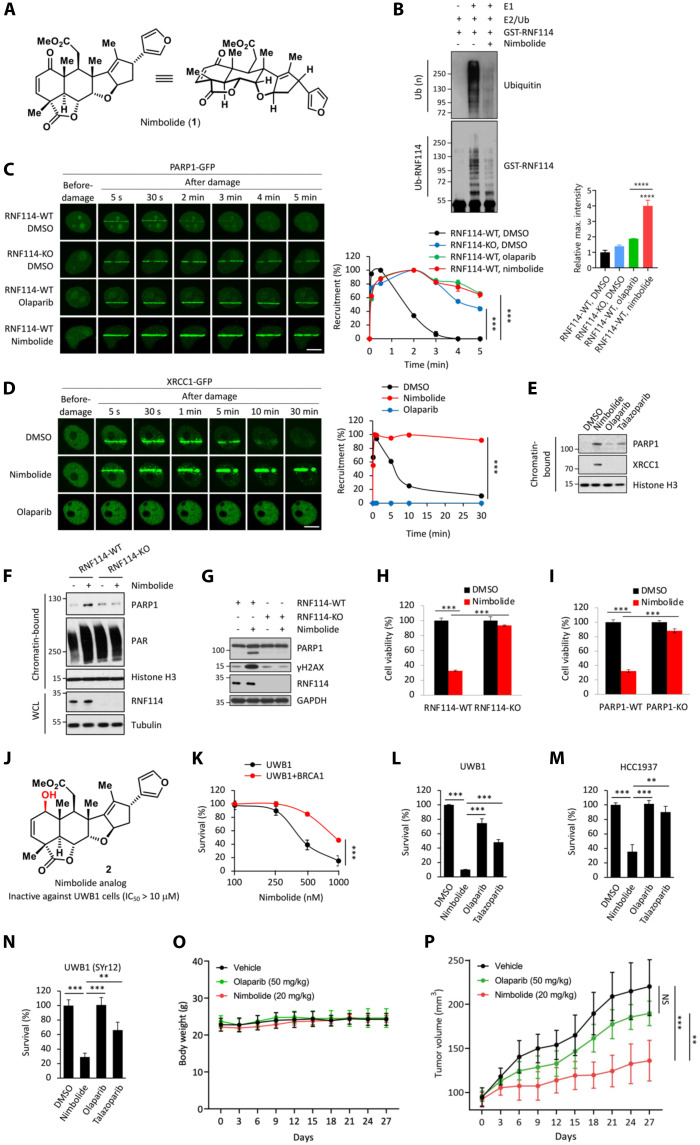
Nimbolide traps PARylated-PARP1 and PAR-dependent DNA repair factors. (**A**) Structure of nimbolide. The total synthesis of nimbolide is reported in (*46*, *47*). (**B**) Nimbolide blocks the auto-ubiquitination of RNF114. (**C**) Nimbolide treatment induces potent PARP1 trapping. The indicated green fluorescent protein (GFP)–tagged PARP1-expressing HeLa cells were pretreated with olaparib (1 μM) or nimbolide (1 μM) for 1 hour. (**D**) Nimbolide, but not olaparib, treatment induces the trapping of XRCC1. HeLa cells expressing GFP-tagged XRCC1 were pretreated with olaparib or nimbolide (1 μM for 1 hour). (**E**) Nimbolide treatment induces potent PARP1 and XRCC1 trapping. HCT116 cells were treated with nimbolide or PARPi (1 μM for 24 hours). (**F**) RNF114 deletion abolishes the nimbolide-induced PARP1 trapping. The indicated HCT116 cells were treated with nimbolide (1 μM for 24 hours). (**G**) RNF114 deletion rescues cells from nimbolide-induced cell death. The indicated HeLa cells were treated with nimbolide (1 μM for 24 hours). (**H** and **I**) RNF114 (H) or PARP1 (I) deletion rescues cells from nimbolide-induced cell death. The indicated HeLa cells were treated with nimbolide (1 μM for 24 hours). The deletion of RNF114 and PARP1 was confirmed as shown in fig. S3H. (**J**) Structure of a nimbolide analog. The compound as shown was inactive against UWB1 cells (IC_50_ > 10 μM). Additional nimbolide analogs are shown in fig. S4. (**K**) Nimbolide treatment is synthetic lethal with respect to *BRCA1* deficiency. UWB1 and UWB1 + BRCA1 cells were subjected to nimbolide treatment for 96 hours. (**L**) Nimbolide induces stronger cytotoxicity in UWB1 cells compared to PARPi. UWB1 cells were treated with nimbolide or PARPi (1 μM for 96 hours). (**M** and **N**) Nimbolide overcomes intrinsic and acquired resistance to PARPi. HCC1937 (M) or UWB1 (SYr12) (N) cells were treated with nimbolide or PARPi (1 μM for 96 hours). (**O** and **P**) Body weight (O) and tumor volume (P) of HCC1937-bearing mice that were treated with vehicle, olaparib, or nimbolide. Scale bars, 10 μm. DMSO, dimethyl sulfoxide.

Nimbolide is a natural product that was originally isolated from the Neem tree (*A. indica*) (Fig. 4A) (*40*). Previous studies suggest that nimbolide has certain anticancer activities, although its underlying MOA is poorly understood (*41*–*44*). Nimbolide was previously shown to covalently modify RNF114 and, in doing so, block the substrate engagement of RNF114 (*45*). Consistent with this model, we found that nimbolide was able to potently block the auto-ubiquitination of RNF114 in an in vitro ubiquitination assay (Fig. 4B and fig. S3G).

Our proteomic and biochemical studies indicate that PARylated-PARP1 is a previously unidentified substrate of RNF114 (Fig. 3). On the basis of these data, we hypothesized that nimbolide could represent a pharmacological approach to manipulating the RNF114-mediated ubiquitination and degradation of PARP1 and, in doing so, to inducing PARP1 trapping. In this case, because nimbolide only occupies the E3 substrate recognition motif, but not the PAR-binding domain, of RNF114 (*45*), we hypothesized that the nimbolide-conjugated RNF114 could functionally mimic its RING domain mutations. Using the laser microirradiation assays, we found that nimbolide treatment resulted in profound PARP1 trapping (Fig. 4C). PARP1 trapping resulting from nimbolide treatment was very similar to that in the RNF114-*RING mutant cells. The kinetics of PARP1 trapping induced by nimbolide was also similar to that of PARPi (Fig. 4C).

Although both PARPi and nimbolide trap PARP1 (Fig. 4C), there is a unique distinction between these two classes of compounds. By blocking PARP1-mediated PARylation, PARPi traps PARP1. In contrast, nimbolide inhibits RNF114 and thereby could result in the trapping of PARylated-PARP1. PAR polymers on PARylated-PARP1 are known to recruit many DNA repair factors (e.g., XRCC1), triggering the formation of a large protein complex involved in the repair of DNA SSBs (*21*). We therefore hypothesize that nimbolide treatment could induce the trapping of not only PARylated-PARP1 but also other PAR-binding DNA repair proteins. To test this hypothesis, we performed laser microirradiation assays and found that, upon sensing DNA strand breaks, XRCC1 was rapidly recruited to the DNA lesions through its PAR-binding domains (Fig. 4D). XRCC1 accumulated and persisted at the DNA lesions in nimbolide-treated cells. In contrast, PARPi treatment blocked the formation of PAR chains, which completely abolished the recruitment of XRCC1 to the DNA lesions (Fig. 4D). Together, these data suggest that by targeting the E3 activity of RNF114 and subsequent proteasomal degradation of PARP1, nimbolide treatment causes potent PARP1 trapping. However, unlike conventional PARPi, nimbolide treatment leads to the trapping of not only PARP1 but also XRCC1.

We also used the chromatin fractionation assay to examine the PARP1 trapping effects of nimbolide and several clinically relevant PARPi (i.e., olaparib and talazoparib). In particular, talazoparib is an FDA-approved PARPi that is thought to be the most potent PARP1 trapper. We found that nimbolide was able to induce more PARP1 trapping compared to olaparib and talazoparib (Fig. 4E). Furthermore, a unique difference between regular PARPi and nimbolide is that by inhibiting PARP1, regular PARPi causes PARP1 trapping. However, nimbolide only inhibits the E3 ubiquitin ligase of RNF114 and likely does not affect the PARylation of PARP1. Consistent with its unique trapping activity of nimbolide (Fig. 4D), we observed that nimbolide, but not the other NAD^+^-competitive PARPi, was able to induce the trapping of XRCC1 (Fig. 4E). We found that nimbolide treatment resulted in profound PARP1 trapping in RNF114-WT cells (Fig. 4F). However, nimbolide-induced PARP1 trapping was abolished in RNF114-KO cells (Fig. 4F). These results therefore indicate that RNF114 is a key mediator of nimbolide-induced PARP1 trapping. Consistent with the role of trapped PARP1 in inducing cytotoxicity, we observed a marked increase of cell death in nimbolide-treated cells. However, these cytotoxic effects of nimbolide were notably decreased in RNF114-KO cells and PARP1-KO cells (Fig. 4, G to I, and fig. S3H). Together, these data suggest that RNF114 is a key mediator of the PARP1-trapping and cytotoxic activity of nimbolide.

Last, we performed additional studies to further validate and probe the pharmacophore of nimbolide. Nimbolide contains a Michael acceptor moiety (Fig. 4A), which enables its covalent conjugation to RNF114 (e.g., via a Cys thiol group) (*45*). In our accompanying reports, we performed the total synthesis of nimbolide via a late-stage fragment-coupling strategy (*46*, *47*). We synthesized several nimbolide analogs where we systemically manipulated this crucial enone moiety. The enone-reduced derivatives allylic alcohol **2** and ketone **3** were successfully obtained (Fig. 4J, fig. S4, and Supplementary Information). Consistent with the model where nimbolide covalently targets RNF114, these analogs (**2** and **3**) completely lost their cytotoxic activity [median inhibitory concentration (IC_50_) > 10 μM] against UWB1 cells (a *BRCA1*^mut^ ovarian cancer cell line; see more discussion below). The lactone opening analogs **4** and **5** (fig. S4) were also found to be inactive. Therefore, we surmise that the enone and lactone moieties form the pharmacophore of nimbolide, which mediates its observed cytotoxic activity.

### Nimbolide treatment is synthetic lethal with *BRCA* mutations

It has been established that *BRCA1/2*-mutated cells are particularly sensitive to PARPi-induced trapping, and these cells are selectively killed by PARPi based on the “synthetic lethality” mechanism (*1*, *48*–*50*). We found that UWB1 cells were highly sensitive to nimbolide (IC_50_ = 0.3 μM). Furthermore, compared to the parental UWB1 cells, UWB1 cells reconstituted with *BRCA1* showed greatly reduced sensitivity to nimbolide (Fig. 4K). Nimbolide showed no toxicity against several other normal cell lines (fig. S5) (*51*). Consistent with the unique trapping activity of nimbolide (which induces the trapping of not only PARP1 but also XRCC1), nimbolide demonstrated superior cytotoxicity in UWB1 cells compared to PARPi (Fig. 4L). These data pointed to the synthetic lethality between nimbolide and *BRCA1* mutations. These results also suggest that *BRCA1* mutations (and potentially mutations of other genes in the homologous repair pathway) will serve as important predictive biomarkers for nimbolide sensitivity.

We also tested whether nimbolide acts synergistically with other DNA-damaging agents, including MMS, doxorubicin, and temozolomide. Compared to nimbolide alone, the combination of nimbolide with these agents showed notably increased toxicity in UWB1 cells (fig. S6A). We also treated UWB1 cells with nimbolide together with AZD6738 [anataxia-telangiectasia and Rad3-related (ATR) inhibitor (*52*)], LY2603618 [a check point kinase 1 (CHK1) inhibitor (*53*)], or SCH900776 [a CHK1 inhibitor (*54*)] (fig. S6B). We found that nimbolide was also able to synergize with these agents to further enhance its cytotoxicity (fig. S6B). Furthermore, PARG is known to be a highly active enzyme that removes the PAR chains from PARP1 and other PARP1 substrates (*55*). By enhancing the PARylation level, PARG inhibitors could further promote the trapping of PAR-dependent DNA repair factors under RNF114-suppressed conditions (e.g., nimbolide-treated cells). We therefore examined the potential synergistic effects between PARG inhibitors and nimbolide. Compared to nimbolide treatment alone, the combination of nimbolide with PDD00017273 [a highly potent PARG inhibitor (*56*–*58*)] showed increased toxicity in UWB1 cells (fig. S7).

### Nimbolide overcomes intrinsic and acquired resistance to PARPi

*BRCA1/2* mutations have been found in tumors originating from many different tissues, including breast, ovary, prostate, and pancreas (*59*). These mutations serve as excellent predictors for PARPi sensitivity. Although several PARPi have been approved for the treatment of breast and/or ovarian cancers with *BRCA* mutations, a substantial fraction of the patients with *BRCA*^mut^ tumors showed de novo resistance, who failed to respond to these agents (intrinsic resistance) (*60*). Because of the superior trapping activity of nimbolide (for both PARP1 and PAR-dependent DNA repair factors), we asked whether nimbolide is able to overcome intrinsic resistance to regular PARPi. HCC1937 is a *BRCA1*^mut^, triple-negative breast cancer cell line that is resistant to PARPi (*61*, *62*). This cell line, however, was exquisitely sensitive to nimbolide even though it was resistant to regular PARPi (i.e., olaparib and talazoparib) (Fig. 4M).

Similar to other targeted therapies, those patients who showed initial response to PARPi often develop resistance, and relapsed disease is commonly observed. Thus, a strategy to overcome PARPi resistance is much needed to improve PARPi to achieve a more complete and durable response in the context of acquired resistance to PARPi. A previous study reported the UWB1 (SYr12) cells, which is a PARPi-resistant UWB1 clone derived from long-term culturing of the parental UWB1 cells in the presence of a PARPi (i.e., olaparib). The resistance mechanism of these cells and the related clones have been ascribed to the transcriptionally rewired DNA damage response network (*62*). We found that UWB1 (SYr12) cells showed sensitivity to nimbolide, but not PARPi (Fig. 4N). Therefore, these data indicate that nimbolide is also able to kill tumors with acquired resistance to PARPi.

On the basis of these results, we further evaluated the in vivo efficacy of nimbolide by using a PARPi-resistant, *BRCA1*^mut^ (HCC1937) tumor xenograft model (Fig. 4, O and P). Consistent with our observations in vitro (Fig. 4N), nimbolide treatment notably suppressed HCC1937 tumor growth in vivo. However, the growth of HCC1937 tumors in vivo remained resistant to olaparib treatment (Fig. 4P). Mice in both treatment groups showed no substantial weight changes (Fig. 4O). Our in vivo data further support the exciting possibility that by inducing potent PARP1 trapping, nimbolide potentially could be useful as a previously unknown class of PARP1-targeting agents for the treatment of *BRCA*^mut^ cancers.

### Nimbolide triggers innate immune response and up-regulates PD-L1 expression

**Fig. 5.**
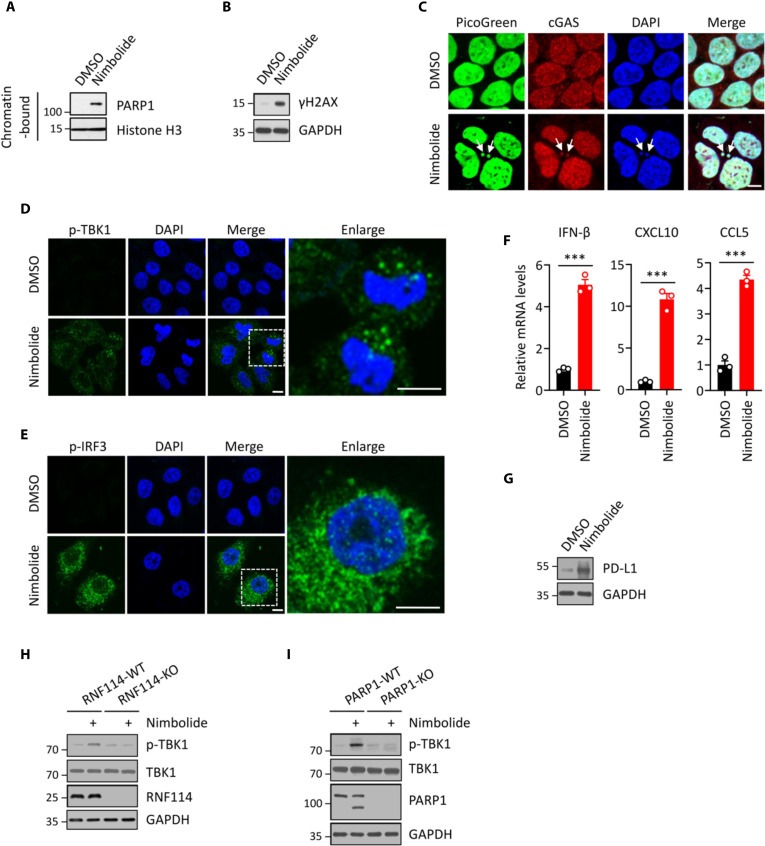
Nimbolide triggers an innate immune response and up-regulates programmed death-ligand 1 (PD-L1) expression. (**A**) Nimbolide treatment induces PARP1 trapping. UWB1 cells were treated with or without nimbolide (1 μM for 48 hours). The chromatin-bound fraction was isolated from these cells and was subject to immunoblotting experiments. (**B**) Nimbolide treatment induces DNA damage response. HeLa cells were treated with or without nimbolide (1 μM for 48 hours). The cell lysates were subject to immunoblotting experiments. (**C**) Nimbolide treatment induces the formation of cytosolic dsDNA and micronuclei. HeLa cells were treated with or without nimbolide (1 μM for 48 hours). Arrows indicate cytosolic dsDNA and micronuclei. (**D**) Nimbolide treatment induces the phosphorylation of TBK1. HeLa cells were treated with or without nimbolide (1 μM for 48 hours). The level of pS172 TBK1 (p-TBK1, green) was detected using the immunofluorescence assay. (**E**) Nimbolide treatment induces the phosphorylation of IRF3. HeLa cells were treated with or without nimbolide (1 μM for 48 hours). The level of pS396 IRF3 (p-IRF3, green) was detected using the immunofluorescence assay. (**F**) Quantitative reverse transcription polymerase chain reaction (qRT-PCR) analyses of IFN-β, CXCL10, or CCL5 in HeLa cells treated with or without nimbolide (1 μM for 48 hours). (**G**) Nimbolide treatment induces the expression of PD-L1. UWB1 cells were treated with or without nimbolide (1 μM for 48 hours). PD-L1 expression was detected using the immunoblot assay. (**H**) RNF114-KO abrogates the nimbolide-induced TBK1 phosphorylation. Control (RNF114-WT) and RNF114-KO HeLa cells were treated with or without nimbolide (1 μM for 48 hours). The whole-cell lysates were subject to immunoblot experiments. (**I**) PARP1-KO abrogates the nimbolide-induced TBK1 phosphorylation. Control (PARP1-WT) and PARP1-KO HeLa cells were treated with or without nimbolide (1 μM for 48 hours). The whole-cell lysates were subjected to immunoblot experiments. Scale bars, 10 μm. cGAS, cyclic guanosine 3′,5′-monophosphate–adenosine 5′-monophosphate synthase; DAPI, 4′ynthase; DAPI, nosine 3′,le; DMSO, dimethyl sulfoxide. pIRF3, phosphorylated IFN regulatory factor 3.

We recently showed that PARPi triggers innate immune signaling by PARP1 trapping-induced DNA damage response (*25*). Because nimbolide induces potent PARP1 trapping and the subsequent DDR (Fig. 5, A and B), we tested whether nimbolide could have any immunomodulatory roles. Immunofluorescence staining experiments showed that nimbolide treatment caused marked accumulation of cytosolic double-stranded DNA (dsDNA) and micronuclei (Fig. 5C). Furthermore, we observed colocalization of cyclic guanosine 3′,5′-monophosphate (GMP)–adenosine 5′-monophosphate (AMP) synthase (cGAS) and cytosolic dsDNA in nimbolide-treated cells (Fig. 5C). cGAS is a critical sensor of cytosolic dsDNA. After the recognition of cytosolic dsDNA, cGAS generates the second messenger cGAMP (cyclic GMP-AMP), which then binds to and activate stimulator of interferon genes (STING). This binding event results in the recruitment and activation of Tank-binding kinase 1 (TBK1). TBK1 phosphorylates a transcription factor interferon (IFN) regulatory factor 3 (IRF3), which leads to its nuclear translocation, and the activation of type I IFN signaling (*63*–*65*).

We found that phosphorylation of TBK1 (p-TBK1), a key downstream effecter of cGAS-STING pathway, was markedly up-regulated in nimbolide-treated cells (Fig. 5D). Furthermore, nimbolide treatment induced the activation and nuclear translocation of p-IRF3, suggesting the activation of cGAS-STING pathway in these cells (Fig. 5E). To further assess the activation of the cGAS-STING pathway, we examined the mRNA levels of a number of downstream target genes in cGAS-STING pathway. The mRNA levels of IFN-β, CXCL10, and CCL5 were markedly increased in nimbolide-treated cells (Fig. 5F). Last, consistent with the superior trapping activity of nimbolide, it was able to induce stronger activation of the cGAS-STING pathway (as shown by higher levels of p-TBK1) compared to other PARPi (i.e., olaparib) (fig. S8A). Collectively, these results demonstrate that nimbolide induces the accumulation of cytosolic dsDNA, which then activates the cGAS-STING-TBK1-IRF3 innate immune signaling.

A critical downstream target of the cGAS-STING pathway is programmed death-ligand 1 (PD-L1), a major ligand of programmed cell death protein-1 (PD-1) (*66*). The binding of PD-L1 to the immune checkpoint molecule PD-1 transmits an inhibitory signal to reduce the proliferation of antigen-specific T cells (*67*). Recent studies suggested that PD-L1 expression is regulated by PARPi (*68*). Because nimbolide treatment induces PARP1 trapping, DNA damage, and innate immune response, we tested whether PD-L1 is also regulated by nimbolide treatment. We found that the expression of PD-L1 was greatly elevated in nimbolide-treated UWB1 cells (Fig. 5G). Consistent with the superior trapping activity of nimbolide, it was able to induce stronger expression of PD-L1 compared to olaparib (fig. S8B). Nimbolide failed to induce TBK1 activation in RNF114-KO or PARP1-KO cells, indicating the specificity of nimbolide in the context of its immunomodulatory roles (Fig. 5, H and I). Together, these data suggest that nimbolide activates the innate immune response and up-regulates PD-L1 expression. These results raise the hypothesis that by inducing the activation of the cGAS-STING pathway, nimbolide could synergize with immune checkpoint inhibitors.

## DISCUSSION

To identify the regulatory factors involved in the PARylation-dependent DNA damage response, we here performed a chromatin localization screen. In this screen, we used quantitative proteomic experiments to identify proteins that become enriched or depleted in the chromatin fraction upon the treatment of genotoxic agents. In response to genotoxic stress, we found that a poorly studied E3 ubiquitin ligase, RNF114, becomes enriched in the chromatin fraction. The recruitment of RNF114 to DNA lesions is mediated by the interaction between PAR chains and the C-terminal PBZ domain of RNF114. Besides the role as a scaffold to recruit RNF114, PAR chains also stimulate the E3 activity of RNF114. Using an IP-MS approach, we subsequently identified PARylated-PARP1 as a previously unknown RNF114 substrate, and RNF114 specifically targeted PARylated-PARP1 for ubiquitin-proteasomal degradation.

Our results suggest that, in response to genotoxic stimuli, PARP1 is recruited to DNA lesions and becomes auto-PARylated. RNF114 is then recruited, in a PAR-dependent manner, to the DNA damage site to catalyze the ubiquitination and subsequent proteasomal degradation of PARylated-PARP1. These results suggest a previously unidentified ubiquitination-dependent mechanism to remove PARylated-PARP1 from DNA lesions. PARPi inhibit the PARylation of PARP1 and thus prevent the recruitment of RNF114 to the DNA lesions. This subsequently blocks the ubiquitination and degradation of PARP1, leading to PARP1 trapping. Consistent with this model, we observed potent PARP1 trapping in cells expressing the RNF114-*RING mutant. The binding of the RNF114-*RING mutant could shield PARylated-PARP1 and therefore prevent its removal by other factors.

**Fig. 6.**
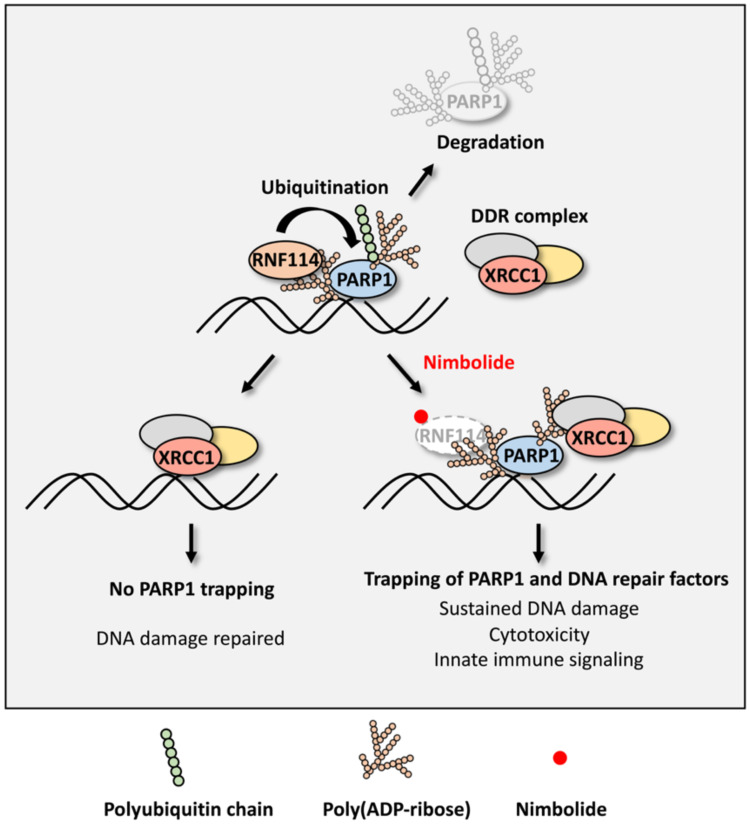
A schematic model of the nimbolide-induced trapping of PARylated-PARP1 and PAR-dependent DNA repair factors.

Nimbolide is a limonoid natural product that is derived from the Neem tree (*40*). Although this compound has previously been shown to have anticancer activity, its exact MOA was poorly characterized (*69*). Using a chemoproteomic approach, a recent study showed that nimbolide covalently modifies RNF114 and, in doing so, prevents its E3 substrate engagement (*45*). We identified, in the current study, PARylated-PARP1 as a previously unknown substrate of RNF114, and blockage of this pathway prevents the ubiquitination and degradation of PARP1 (Fig. 6). On the basis of these findings, we hypothesized that by targeting RNF114, nimbolide treatment could mimic the RNF114-*RING mutant, which serves as a pharmacological approach to regulate the degradation of PARP1. Consistent with this hypothesis, we observed potent PARP1 trapping in nimbolide-treated cells.

In the context of PARP1 removal from the chromatin, RNF114-mediated degradation is likely complementary with several recently described models (*70*–*72*). Although both PARPi and nimbolide induce PARP1 trapping, a key difference between these two classes of compounds is that PARPi traps PARP1, whereas nimbolide likely traps PARylated-PARP1 (Fig. 6). In this case, PARP1 inhibition and PARP1 trapping are coupled for these PARPi, i.e., PARPi cause PARP1 trapping by blocking its auto-PARylation and also by allosterically enhancing its DNA binding (*73*). In contrast, nimbolide treatment could potentially decouple PARP1 trapping from PARP1 inhibition. Specifically, RNF114 inhibition likely prevents the removal of PARylated-PARP1 from DNA lesions (Fig. 6). Protein-linked PAR polymers are known to recruit many proteins that bear PAR-binding domains. As an example, in response to genotoxic stress, PAR polymers function as a scaffold to recruit a protein called XRCC1, triggering the formation of a large protein complex involved in the repair of DNA SSBs (*21*). We found that PARPi treatment completely abolished the recruitment of XRCC1 to DNA lesions, whereas potent retention of XRCC1 was observed in nimbolide-treated cells. These data again suggest that RNF114 inhibition impairs the removal of PARylated-PARP1 from the DNA damage site, leading to the subsequent retention of PARP1, XRCC1, and, likely, other PAR-dependent DNA repair factors.

Although the various FDA-approved PARPi inhibit the catalytic activity of PARP1 with similar potency, they differ in their allosteric binding to PARP1, leading to the unequal PARP1-trapping activities (*73*). It has been shown that DDR, cytotoxicity, and innate immune responses in PARPi-treated cells are all positively correlated with the degree of PARP1 trapping induced by the specific PARPi (*22*, *25*–*28*). Because nimbolide traps not only PARP1 but also XRCC1, we then examined the anticancer activity of nimbolide (Fig. 6). First, we found that, similar to other PARPi, nimbolide treatment is synthetically lethal with respect to *BRCA1* mutations. Specifically, we showed that nimbolide demonstrated enhanced cytotoxicity against UWB1 cells compared to UWB1 cells reconstituted with *BRCA1* (Fig. 4K). These results suggest that mutations of *BRCA1* (and likely *BRCA2* and other genes in the HR pathway) will serve as important predictive biomarkers for nimbolide sensitivity. Second, we showed that the unique trapping activity of nimbolide can be translated into its distinct advantages over PARPi in overcoming PARPi resistance (both intrinsic and acquired resistance). Specifically, we showed that both HCC1937 (a *BRCA1*^mut^ breast cancer cell line that is intrinsically resistant to regular PARPi) and UWB1 (SYr12) (a *BRCA1*^mut^ ovarian cancer cell line that has developed acquired resistance to PARPi) remain exquisitely sensitive to nimbolide (Fig. 4, M and N). Using a PARPi-resistant, *BRCA1*^mut^ (HCC1937) tumor xenograft model, we demonstrated that nimbolide could potentially overcome PARPi resistance of *BRCA*-mutant tumors in vivo (Fig. 4, O and P). Third, nimbolide was able to act synergistically with DNA-damaging agents and DNA repair machinery inhibitors (e.g., ATR and CHK1 inhibitors) (fig. S7, A and B). We showed that by inducing PARP1 trapping and DDR, nimbolide triggers the activation of innate immune signaling. This leads to the elevated expression of immunomodulatory proteins, including PD-L1.

Collectively, we showed that enhanced trapping of PARP1 and PARylated DNA repair factors induced by nimbolide treatment can be translated into distinct advantages compared to NAD^+^-competitive PARPi. The full therapeutic potential of nimbolide and its analogs as a monotherapy, as well as its combination with other DNA-damaging agents and immune checkpoint inhibitors, warrants future studies.

In summary, our studies suggest that in response to genotoxic stress, PARP1 is recruited to DNA lesions and becomes auto-PARylated (Fig. 6). RNF114 is then recruited, in a PAR-dependent manner, to the DNA damage site to catalyze the ubiquitination and subsequent proteasomal degradation of PARP1. By targeting the substrate recognition domain of RNF114, nimbolide treatment blocks PARP1 degradation, leading to PARP1 trapping. Unlike regular PARPi, nimbolide treatment induces the trapping of not only PARylated-PARP1 but also PAR-dependent DNA repair factors. We then showed that nimbolide is synthetic lethal with respect to *BRCA* mutations, and it was able to kill cancer cells with intrinsic and acquired resistance to PARPi. Last, we demonstrated that nimbolide treatment is synergistic with other DNA damaging agents, activates innate immune response, and up-regulates PD-L1 expression. Our results point to the exciting possibility of therapeutically targeting the RNF114-mediated PARP1 trapping pathway by nimbolide and its analogs (*46*, *47*) for the treatment of *BRCA*^mut^ cancers.

## MATERIALS AND METHODS

### Materials

The antibodies used in this study were obtained from commercial sources, including anti-PAR (Trevigen, 4335-MC-100), anti–glyceraldehyde-3-phosphate dehydrogenase (GAPDH) (Thermo Fisher Scientific, MA1-16757), anti–Flag-tag (Thermo Fisher Scientific, MA1-91878), anti–glutathione *S*-transferase (GST) [Cell Signaling Technology (CST), 2622S], anti-RNF114 (Santa Cruz Biotechnology, sc-514747), anti-histone (CST, 4499S), anti-ubiquitin (Santa Cruz Biotechnology, sc-8017), anti-PARP1 (CST, 9542S), anti-γH2AX (CST, 9718S), anti–p-TBK1 (CST, 5483S), anti-TBK1 (CST, 38066S), anti–p-IRF3 (CST, 29047S), anti-IRF3 (CST, 11904S), anti-cGAS (CST, 15102S), anti-STING (CST, 13647S), and anti–PD-L1 (CST; 13684S). Nimbolide was purchased from Sigma-Aldrich (SMB00586). The PARP inhibitors rucaparib (Selleck Chemicals, S4948), olaparib (Selleck Chemicals, S1060), and talazoparib (Selleck Chemicals, S7048) were purchased from the indicated sources. All other reagents including MG132 (Selleck Chemicals, S2619), H_2_O_2_ (Thermo Fisher Scientific, H325), MMS (Sigma-Aldrich, 129925), doxorubicin (Sigma-Aldrich, D1515), temozolomide (Sigma-Aldrich, T2577), AZD6738 (Cayman Chemical, 21035), LY2603618 (Apexbio, A8638), and SCH900776 (Cayman Chemical, 18131) were obtained from commercial sources.

### Cell culture

All the cells were purchased from American Type Culture Collection (ATCC) and cultured according to the directions from ATCC. HCT116 and HeLa cells were maintained in the high-glucose Dulbecco’s modified Eagle’s medium (DMEM) supplemented with 10% fetal bovine serum (FBS). HCC1937 cells were maintained in RPMI 1640 (ATCC) supplemented with 15% FBS. UWB1.289 (UWB1), UWB1 + BRCA1, and UWB1 (SYr12) cells were gifts from L. Zou (Harvard Medical School). UWB1 and UWB1 + BRCA1 cells were maintained in RPMI 1640 (ATCC) and mammary epithelial cell growth medium (MEGM) bullet kit (1:1; Lonza) with 3% FBS. UWB1 (SYr12) cells were maintained in RPMI 1640 and MEGM bullet kit with 3% FBS and 1 μM PARPi (olaparib, Selleck Chemicals) (*62*).

### Plasmid and construction

The RNF114 clone was obtained from the Center for Human Growth and Development of UTSW. The RNF114 cDNA was subcloned into the pcDNA3 (Addgene), pCDNA5-ZZ-TEV-Flag vector (Addgene), or pGEX-4T-3 (Addgene) vectors for transient transfections. Besides, the RNF114 cDNA was transferred into the plenti-6.3-V5-Dest vector (Thermo Fisher Scientific) for the subsequent construction of stable cell lines. Various site mutations of RNF114 were introduced using standard site-directed mutagenesis techniques. RNF114 CRISPR KO constructs were made according to a previously described protocol (*74*). Basically, three top ranked single guide RNAs were chosen (RNF114 sg1F: CACCGGTGTACGAGAAGCCGGTAC; RNF114 sg1R: AAACGTACCGGCTTCTCGTACACC; RNF114 sg2F: CACCGGACACGTGAAGCGTCCTAG; RNF114 sg2R: AAACCTAGGACGCTTCACGTGTCC; RNF114 sg3F: CACCGCACGTGTCCCGTGTGCTTAG; RNF114 sg3R: AAACCTAAGCACACGGGACACGTGC), and they were subcloned into *LentiCRISPR* v2 vector (Addgene). All the mutant constructs were confirmed by DNA sequencing analysis. PARP1–green fluorescent protein (GFP) and XRCC1-GFP plasmids were gifts from X. Yu (City of Hope).

### Construction of stable cell lines

The plenti or pLKO.1 construct (8 μg), VSVG (6 μg), and delta8.9 (6 μg) were cotransfected into human embryonic kidney (HEK) 293TD cells in 10-cm dishes with Lipofectamine 2000 (Sigma-Aldrich). The medium was changed 6 hours after transfection. Viruses were collected twice at 24 and 48 hours after transfection, respectively, and then combined together. Subsequently, 3 ml of virus was added to each well of HCT116 or HeLa cells in six-well plates with polybrene (8 μg/ml). After splitting the cells once, HCT116 or HeLa cells were infected with the previously collected virus again using the same procedure. The culture medium was replaced after 48 hours with a fresh growth medium containing blasticidin (2 μg/ml) or puromycin. To generate the RNF114 CRISPR KO cell lines, we performed single-cell clone selection after recombinant lentiviruses infection (the viruses were produced by transfecting all three *LentiCRISPR* v2-sgRNF114 plasmids together). The clones that were completely depleted of the RNF114 protein were chosen for future experiments.

### Sample preparation and the MS-based chromatin relocalization screen

To analyze how chromatin proteins respond to genotoxic stress, HCT116 cells were pretreated with talazoparib (1 μM for 1 hour), which was followed by the treatment with H_2_O_2_ (2 mM for 5 min) or MMS (0.01% for 1 hour) as indicated. Cells were washed with cold 1× phosphate-buffered saline (PBS) and the chromatin-bound proteins were extracted using the subcellular fractionation kit (Thermo Fisher Scientific). Protein concentrations were measured by the bicinchoninic acid (BCA) Assay Kit (Thermo Fisher Scientific). For the TMT experiments, proteins were reduced with dithiothreitol (2 mM for 10 min) and alkylated with iodoacetamide (50 mM for 30 min) in the dark. Proteins were then extracted by methanol/chloroform precipitation and were washed by ice-cold methanol. Protein pellets were redissolved in 400 μl of an 8 M freshly prepared urea buffer [50 mM tris-HCl and 10 mM EDTA (pH 7.5)]. The proteins were digested by Lys-C at a 1:100 enzyme/protein ratio for 2 hours at room temperature (RT), followed by trypsin digestion at a 1:100 enzyme/protein ratio overnight at RT. Peptides were desalted with Oasis HLB cartridges and were resuspended in 200 mM Hepes (pH 8.5). For each sample, 100 μg of peptides was reacted with the corresponding amine-based TMT six-plex reagents (Thermo Fisher Scientific) for 1 hour at RT. The labeling scheme was as follows: 126: control, 127: H_2_O_2_, 128: MMS, 129: talazoparib, 130: talazoparib and H_2_O_2_ and 131: talazoparib and MMS. These actions were quenched with a hydroxylamine solution, and the peptide samples were combined.

The TMT samples were desalted and were fractionated by basic pH-reversed phase HPLC on a ZORBAX 300Extend-C18 column. Buffer A was 10 mM ammonium formate in water (pH 10.0). A gradient was developed at a flow rate of 0.2 ml/min from 0 to 70% buffer B [1 mM ammonium formate (pH 10.0) and 90% acetonitrile]. Seventeen fractions were collected, which were lyophilized, desalted, and analyzed by LC-MS/MS experiments as described previously (*27*, *30*, *75*).

### Laser microirradiation assays

Cells grown on 35-mm glass-bottomed culture dishes (Mattek) were transfected with the GFP-tagged RNF114, GFP-PARP1, or GFP-XRCC1 plasmid for 24 hours. After the compound treatment as indicated in each experiment, laser microirradiation was performed using a Zeiss LSM 780 inverted confocal microscope coupled with the MicroPoint laser illumination and ablation system (Photonic Instruments). The GFP fluorescence at the laser line was recorded at the indicated time points and was then analyzed with the ImageJ software. The level of PARP1 trapping (relative max intensity) was quantified by dividing the GFP signal from trapped PARP1 (trapped PARP1-GFP, combined across the different time points) by the total GFP signal in the nucleus (total PARP1-GFP, combined across the different time points). The level of PARP1 trapping for the different RNF114 conditions was then normalized to that in the RNF114-WT sample.

### Immunoblot analysis

Cells were collected and washed once with cold 1× PBS. Then, cells were lysed with the 1% SDS lysis buffer [1% SDS, 10 mM Hepes (pH 7.0), 2 mM MgCl_2_, and 500 U of universal nuclease]. Protein concentrations were measured by the BCA Assay Kit (Thermo Fisher Scientific). The same amount of protein was loaded onto an SDS–polyacrylamide gel electrophoresis (SDS-PAGE) gel. After electrophoretic separation, proteins were transferred to a NC (nitrocellulose) membrane (GE Healthcare). For the dot immunoblot analysis, samples were loaded directly to the NC membrane. The membrane was blocked and was then blotted with the primary antibodies overnight at 4°C, which was followed by incubation with the secondary antibody for 1 hour at RT. The blots were developed using enhanced chemiluminescence and were exposed on autoradiograph films.

### Co-immunoprecipitation analysis

Cells were collected and lysed in the IP lysis buffer [50 mM Tris (pH 7.4), 1 mM EDTA, 150 mM NaCl, 0.5% NP-40, and 1× protease inhibitor cocktail]. After incubation for 1 hour at 4°C, cell lysates were centrifuged at 14,000*g* for 10 min at 4°C. The supernatants were transferred and incubated with the corresponding agarose beads overnight at 4°C. The beads were washed three times with the IP wash buffer, and the immunocomplexes were eluted from the beads by boiling at 95°C for 30 min and were subjected to immunoblot analysis. The tandem affinity purification (TAP)-IP-MS experiments were performed according to a previously described protocol (*76*).

### Immunofluorescence microscopy

After the treatment with DMSO or nimbolide, HeLa cells were washed once with 1× PBS and then fixed with 4% paraformaldehyde for 20 min at RT, followed by three times wash with 1× PBS. The cells were permeabilized with 0.25% Triton X-100 in 1× PBS for 10 min and blocked with 1× PBS containing 2% bovine serum albumin for 1 hour. Fixed cells were incubated with primary antibodies at 4°C overnight, followed by the incubation with the fluorescent secondary antibody for 1 hour at RT. Cells were washed three times with 1× PBS for 5 min and stained with 4′,6-diamidino-2-phenylindole (DAPI) (Thermo Fisher Scientific) for 2 min. DAPI was used to visualize the nuclei. Cells were washed with 1× PBS and mounted with the FluorSave reagent (Millipore). The fluorescence images were then collected with a Zeiss LSM 880 Airyscan inverted confocal microscope.

### Xenograft mouse models

All animal experiments were performed in compliance with the Institutional Animal Care and Use Committee at the Columbia University Irving Medical Center. Female NOD scid gamma (NSG; NOD-SCID) mice (the Jackson Laboratory) at 6 to 8 weeks of age were used. Tumors were engrafted in NSG mice by subcutaneous injection of 1 × 10^7^ cells of HCC1937 in RPMI 1640 medium supplemented with 50% Matrigel (BD Biosciences). Ten days after the injection, mice carrying ~100 mm^3^ subcutaneous tumors were assigned randomly to control and various treatment groups (*n* = 6 to 7 for each group). Tumor-bearing mice were intraperitoneally (i.p.) injected with either vehicle, olaparib (50 mg/kg), or nimbolide (20 mg/kg) daily. The weight of the mice was monitored every 3 days, and tumor volume was also measured with calipers every 3 days. Tumor volumes were calculated using a modified ellipsoid formula: Tumor volume = ½(length × width^2^).

### Quantitative reverse transcription polymerase chain reaction

The cells with the treatment of DMSO or nimbolide were lysed with TRIzol (Thermo Fisher Scientific). Then, the total RNA was extracted according to the manufacturer’s protocol and subject to reverse transcription with SuperScript III One-Step RT-PCR System (Thermo Fisher). The quantitative reverse transcription polymerase chain reaction (qRT-PCR) experiments were performed with a Power SYBR Green PCR Master Mix (Thermo Fisher Scientific) and specific primers listed as follows: GAPDH (sense: ACAACTTTGGCATTGTGGAA; anti-sense: GATGCAGGGATGATGTTCTG), IFN-β (sense: AGCTGAAGCAGTTCCAGAAG; anti-sense: AGTCTCATTCCAGCCAGTGC), CXCL10 (sense: GGCCATCAAGAATTTACTGAAAGCA; anti-sense: TCTGTGTGGTCCATCCTTGGAA), and CCL5 (sense: ATCCTCATTGCTACTGCCCTC; anti-sense: GCCACTGGTGTAGAAATACTCC).

### In vitro PARylation assays

PARP1 (500 ng; Tulip Biolabs), sheared Salmon Sperm DNA (100 ng; Thermo Fisher Scientific), and NAD^+^ (500 μM) were incubated in the reaction buffer [50 mM tris (pH 7.5), 4 mM MgCl_2_, 20 mM NaCl, and 250 μM dithiothreitol (DTT)] at RT for 1 hour. Reactions were terminated by the SDS loading buffer and the samples were subject to immunoblot analyses by an anti-PAR antibody.

### In vitro ubiquitination assays

Recombinant GST-RNF114 was purified from isopropyl-β-d-thiogalactopyranoside-induced *Escherichia coli* and confirmed with Coomassie-stained SDS-PAGE. Flag-RNF114-WT, Flag-RNF114-*PBZ mutant, Flag-RNF114-*RING mutant, or hemagglutinin-CHIP protein was purified from HEK293T cells transfected with RNF114-WT, RNF114-*PBZ mutant, RNF114-*RING mutant, or CHIP plasmids. To measure the auto-ubiquitination of RNF114, UBE1 (50 nM; Boston Biochem), UBE2D1 (50 nM; Boston Biochem), and ubiquitin (200 μM; Boston Biochem) were incubated with the recombinant RNF114 (1 μg) at 37°C in the ubiquitination reaction buffer [50 mM Tris-HCl (pH 7.5), 2.5 mM MgCl_2_, 2 mM DTT, and 2 mM adenosine 5′-triphosphate)]. Reactions were terminated by SDS loading buffer and were boiled. The supernatant samples were subject to immunoblot analysis by an anti-ubiquitin antibody. For the in vitro PARylated-PARP1 ubiquitination assays, PARP1 or PARylated-PARP1 (350 ng) was incubated together with UBE1 (50 nM; Boston Biochem), UBE2D1 (50 nM; Boston Biochem), ubiquitin (200 μM; Boston Biochem), and recombinant RNF114 (WT or mutants) (1 μg) at 37°C in the ubiquitination reaction buffer as above. After the in vitro ubiquitination reaction, the samples were denatured by the addition of 1% SDS (final concentration) and were boiled. The samples were diluted (10×) using the lysis buffer (to reduce the concentration of SDS to 0.1%), and were subjected to IP using the PARP1 antibody (to remove the interference from RNF114). The isolated PARP1 was probed using the anti-ubiquitin antibody. To measure the auto-ubiquitination of RNF114 suppression by nimbolide, GST-RNF114 was incubated with E1/E2/ubiquitin in the presence of nimbolide (1 μM).

### Colony formation assays

HCT116 control (RNF114-WT) or RNF114-KO cells were seeded into 6-cm dishes (about 1000 cells per dish). The cells were treated with or without H_2_O_2_ (2 mM for 5 min) and followed by 14 days culture. The viable cells were fixed by methanol and were stained with crystal violet.

### Cell viability measurement

Cells were plated into 96-well plates at densities of 1000 cells per well. Next day, cells were treated as indicated. Cell viability was measured using the CellTiter-Glo assay (Promega) according to the manufacturer’s instructions. Briefly, after incubation, RT CellTiter-Glo reagent was added 1:1 to each well, and the plates were incubated at RT for 2 min. Luminescence was measured with the Synergy HT Multi-Detection Microplate Reader and was normalized against control cells.

### Statistics

All of the statistical analyses (*t* tests) were performed using the GraphPad Prism software (v.8). Data were derived from the average of three biological replicate experiments and were presented as the means ± SEM. *P < 0.05, **P < 0.01, ***P < 0.001, ****P < 0.0001, and NS, not significant.

## Supplementary Materials

### This PDF file includes:

Figs. S1 to S8 Supplementary information for synthetic procedures Legend for table S1
