# Supplementary material for: Nimbolide targets RNF114 to induce the trapping of PARP1 and synthetic lethality in BRCA-mutated cancer
Source: Sci Adv. 2023 Oct 25;9(43):eadg7752. doi: 10.1126/sciadv.adg7752 (PMC10599614; doi:10.1126/sciadv.adg7752)
Supplement: Supplementary file 1 — Figs. S1 to S8 Supplementary information for synthetic procedures Legend for table S1 [file sciadv.adg7752_sm.pdf]

Science Advances
AAAS

Supplementary Materials for

Nimbolide targets RNF114 to induce the trapping of PARP1 and synthetic lethality in *BRCA*-mutated cancer

Peng Li *et al.*

Corresponding author: Yonghao Yu, yy3213@cumc.columbia.edu

Sci. Adv. **9**, eadg7752 (2023)
DOI: 10.1126/sciadv.adg7752

The PDF file includes:

Figs. S1 to S8
Supplementary information for synthetic procedures
Legend for table S1

Other Supplementary Material for this manuscript includes the following:

Table S1

Supplementary figures:

fig. S1.

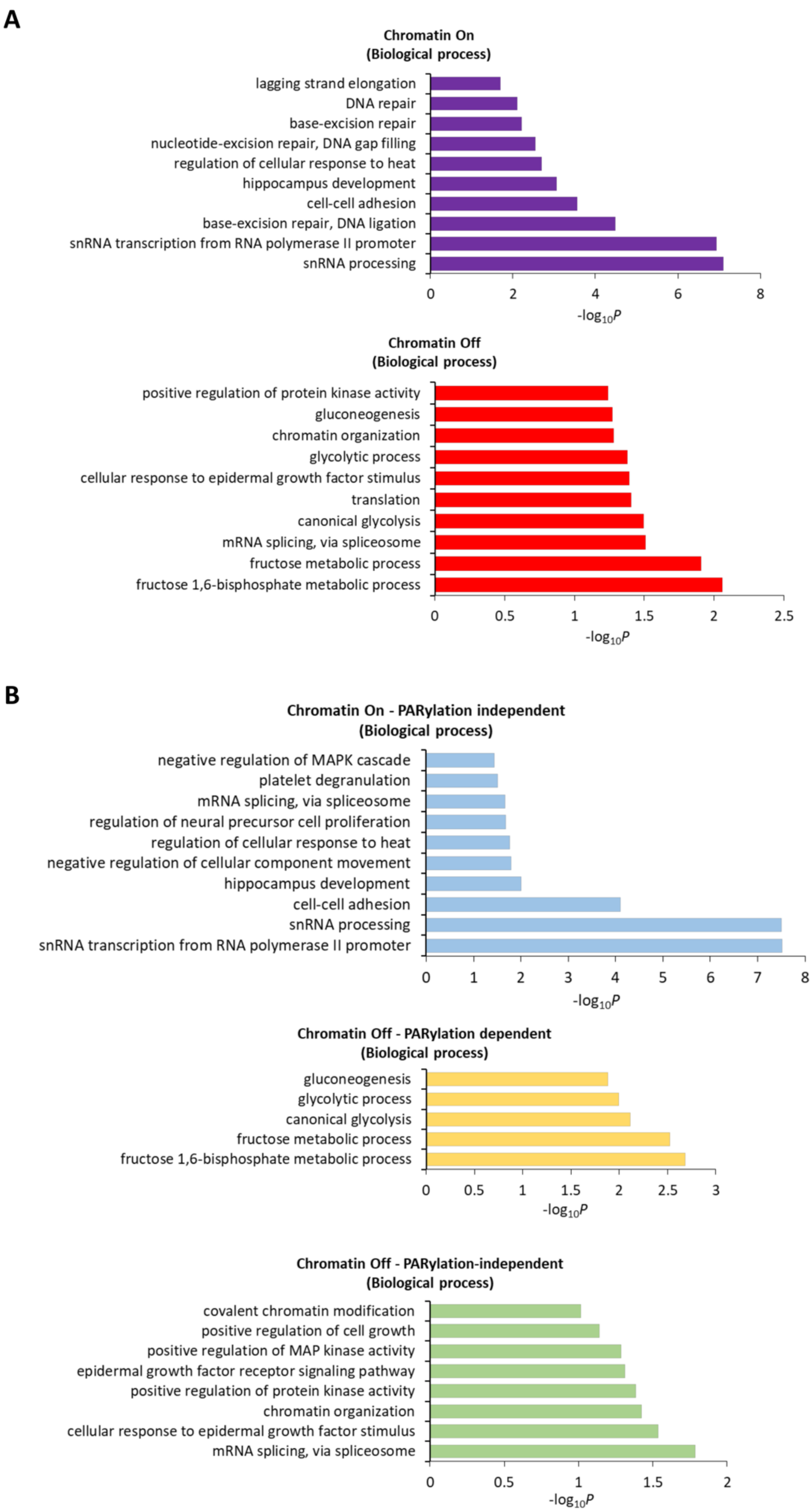

fig. S1. GO analysis of the Chromatin-On and -Off proteins. (**A**) GO analysis of the Chromatin-On and -Off proteins. These proteins include all the proteins (i.e., PARylation-dependent and PARylation-independent). (**B**) GO analysis of the Chromatin-On-PARylation independent, Chromatin-Off-PARylation dependent, and Chromatin-Off-PARylation independent proteins.

fig. S2.

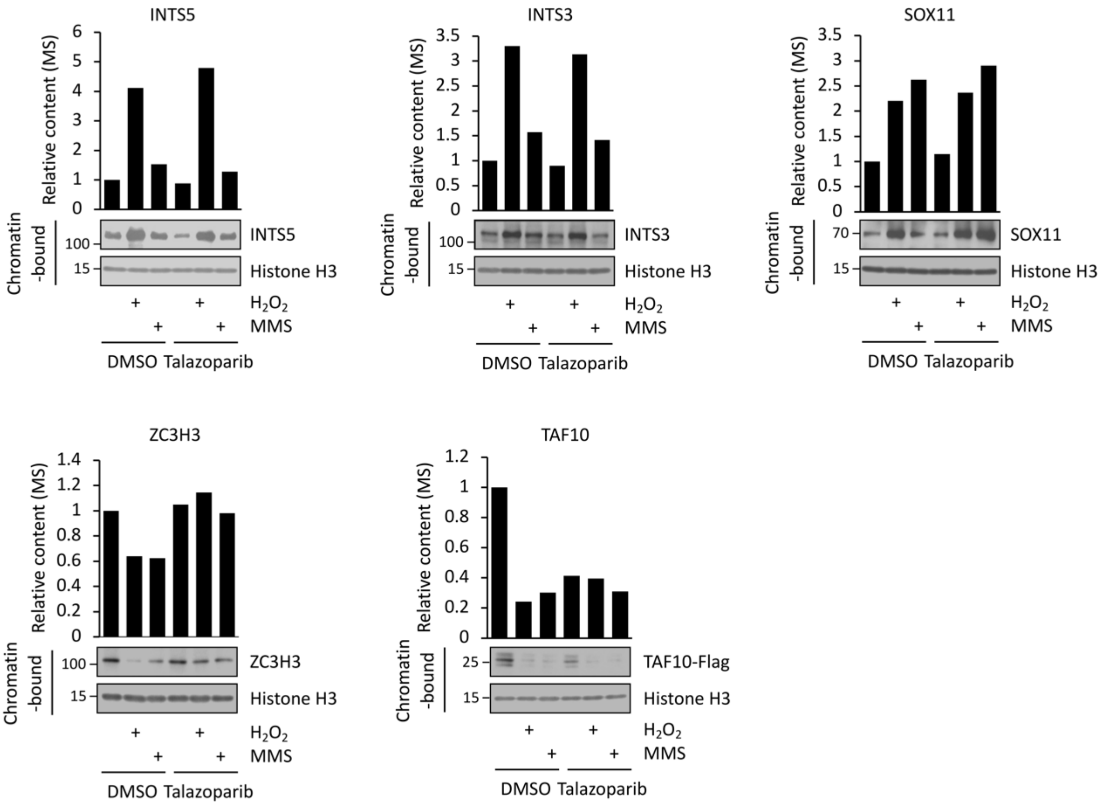

fig. S2. Validation of DDR-induced re-localization events. The abundance of INTS5, INTS3, SOX11, ZC3H3, and TAF10 in the chromatin fraction as measured in the proteomic (upper panel) and immunoblot experiments (lower panel).

fig. S3.

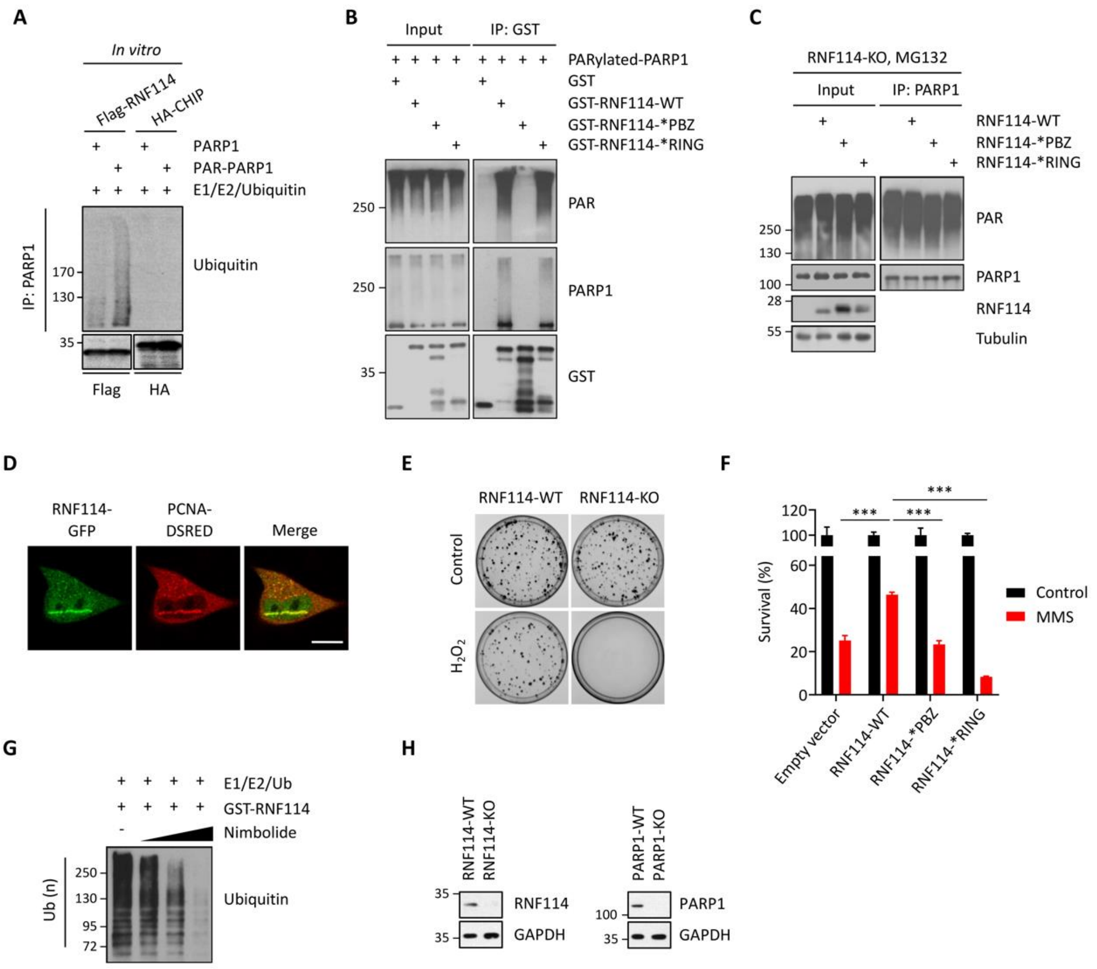

fig. S3. RNF114 targets PARylated-PARP1 for ubiquitin-proteasomal degradation. (**A**) *In vitro* ubiquitination assays of PARP1 or PARylated-PARP1. The assay was performed using the protocol as shown in Fig. 3B, with PARP1 or PARylated-PARP1 (both were used at 1 μg) as the potential substrate. Purified RNF114 or CHIP was subject to *in vitro* ubiquitination experiments in the presence of PARP1 or PARylated-PARP1. After the *in vitro* ubiquitination reaction, the samples were denatured by the addition of 1% SDS (final concentration) and were boiled. The samples were diluted (10X) using the lysis buffer (to reduce the concentration of SDS to 0.1%), and were subject to immunoprecipitation using the PARP1 antibody (to remove the interference from RNF114 or CHIP). The isolated PARP1 was probed using the anti-ubiquitin antibody. (**B**) RNF114 interacts with PAR. Immunoblot analyses of the interaction between PARylated-PARP1 and RNF114 *in vitro*. The recombinant GST-RNF114-WT, GST-RNF114-*PBZ mutant, or GST-RNF114-*RING mutant was

incubated with PARylated-PARP1. The samples were subject to glutathione-based enrichment (for the isolation of GST and GST-fusion proteins). (**C**) PARylated-PARP1 is not degraded by RNF114 in proteasome inhibition. RNF114-KO cells were reconstituted with RNF114-WT, RNF114-*PBZ mutant, or RNF114-*RING mutant. These cells were pre-treated with MG132 (10 μM for 6 h) and were then treated with H_2O_2 (2 mM for 5 min). PARP1 was isolated using immunoprecipitation and was subject to immunoblot analyses using the indicated antibodies. (**D**) RNF114 is co-localized with PCNA during DNA damage response. Staining of RNF114-GFP (Green) and PCNA-DSRED (Red) during laser microirradiation-induced DNA damage. Scale Bars, 10 μm. (**E**) RNF114 is involved in DNA damage response. Control (RNF114-WT) and RNF114-KO HCT116 cells were treated with or without H_2O_2 (2 mM for 5 min). Cell viability was measured using the colony formation assay. (**F**) Mutation of the RING domain in RNF114 renders cells susceptible to genotoxic stress. RNF114-KO HCT116 cells were reconstituted with RNF114-WT, RNF114-*PBZ mutant, or RNF114-*RING mutant. The cells were treated with MMS (1 mM for 9 h). Cell viability was measured using the CellTiter-Glo assay. (**G**) Nimbolide blocks the auto-ubiquitination of RNF114 in an *in vitro* ubiquitination assay. Where indicated, GST-RNF114 was incubated with E1/E2/ubiquitin in the presence of increasing concentrations of nimbolide (0.1 μM, 0.5 μM or 1 μM). (**H**) Generation of the RNF114-KO and PARP1-KO HeLa cells. RNF114 or PARP1 was deleted in HeLa cells using the CRISPR-Cas9 system. Whole cell lysates were subject to immunoblot analyses using the indicated antibodies.

fig. S4.

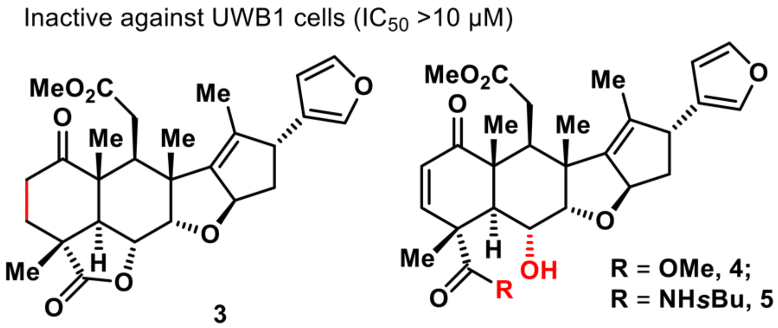

fig. S4. Structure of the various nimbolide analogs. These compounds were inactive against UWB1 cells.

fig. S5.

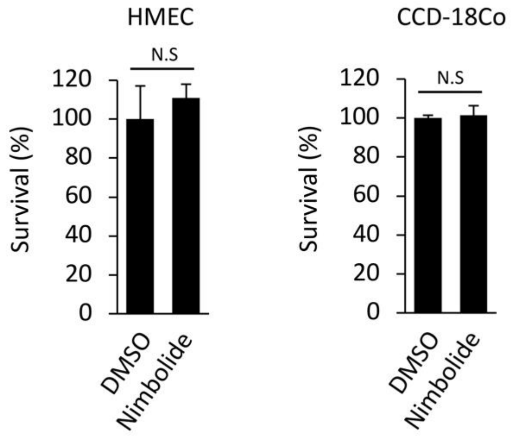

fig. S5. The primary and *BRCA1*-WT cells are not sensitive to nimbolide. HMEC and CCD-18Co cells were treated with or without nimbolide (1 μM). Cell viability was measured using a CellTiter-Glo assay.

fig. S6.

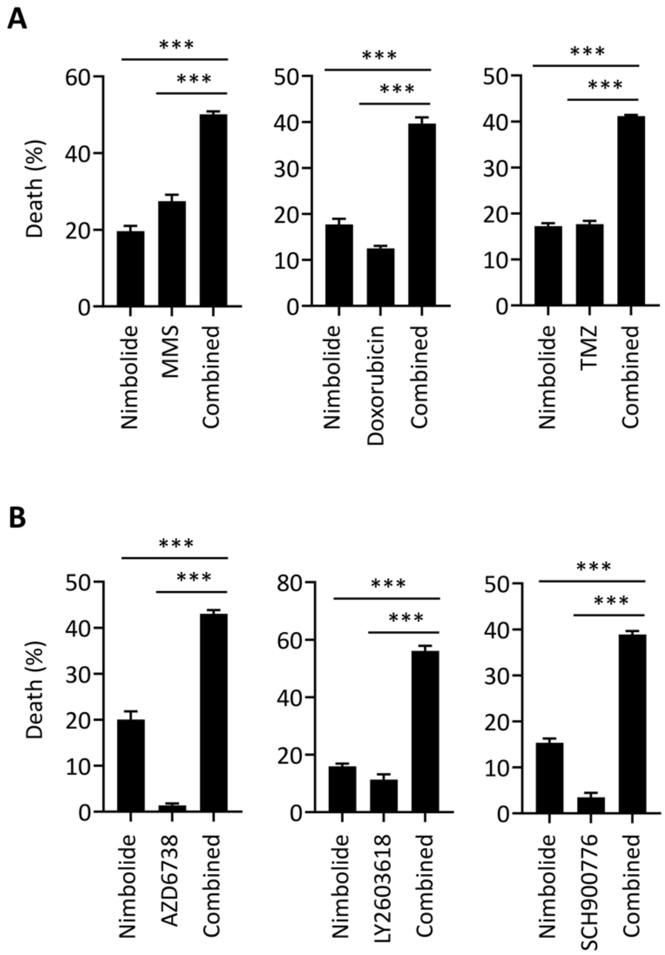

fig. S6. Nimbolide synergizes with DNA damaging agents. (**A**) The synergistic effect between nimbolide and DNA damaging agents. UWB1 cells were treated with either nimbolide (0.25 μM) alone, or nimbolide in combination with various DNA-damaging agents (MMS (10 μM), Doxorubicin (0.01 μM), and Temozolomide (TMZ, 10 μM)) for 96 hrs. (**B**) The synergistic effects between nimbolide and inhibitors of the DNA damage repair machinery. UWB1 cells treated with either nimbolide (0.25 μM) alone, or nimbolide in combination with AZD6738 (0.1 μM), LY2603618 (0.1 μM), or SCH900776 (0.1 μM)) for 96 hrs.

fig. S7.

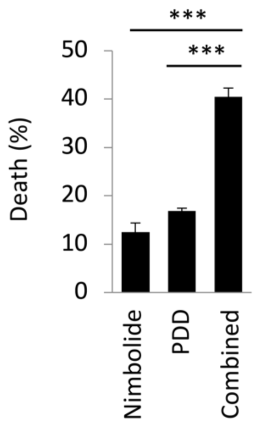

fig. S7. Nimbolide synergizes with a PARG inhibitor. The synergistic effects between nimbolide and PDD00017273 (a PARG inhibitor, PDD). UWB1 cells were treated with either nimbolide (0.25 μM) alone, PDD00017273 (0.25 μM) alone or nimbolide in combination with PDD00017273 (both at 0.25 μM) for 96 hrs.

fig. S8.

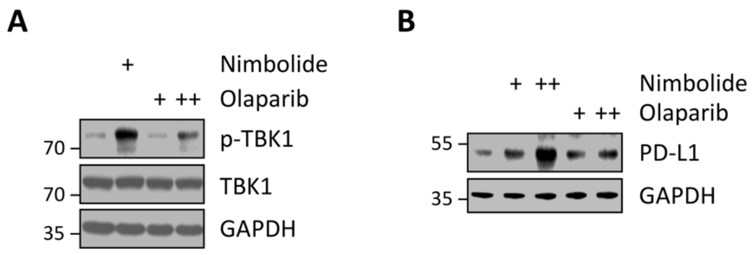

fig. S8. Nimbolide treatment induces the activation of innate immune signaling. (**A**) Nimbolide induces strong activation of innate immune signaling. HeLa cells were treated with nimbolide (1 μM, +) or Olaparib (1 μM, + or 10 μM, ++) for 48 h. The whole cell lysates were subject to immunoblot experiments using the indicated antibodies. (**B**) nimbolide induces expression of PD-L1. UWB1 cells were treated with nimbolide (1 μM, + or 2 μM, ++) or Olaparib (5 μM, + or 10 μM, ++) for 48 hours. The whole cell lysates were subject to immunoblot experiments using the indicated antibodies.

Supplementary information for synthetic procedures:

Compound 3

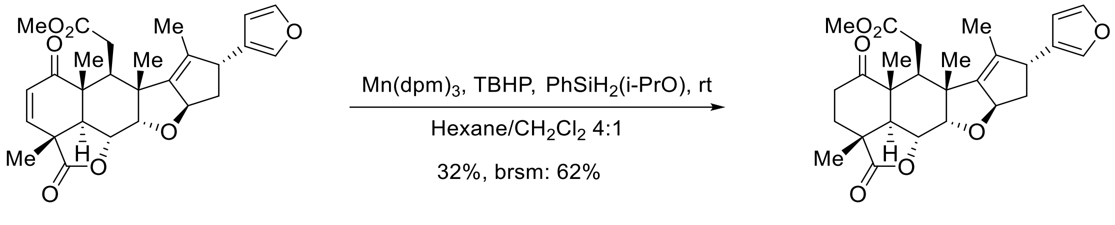

1 3

Nimbolide (20 mg, 0.0429 mmol) and $Mn(dpm)_3$ (1.3 mg, 0.0021 mmol, 5 mol%) were dissolved in 2 mL hexane and 0.5mL CH_2Cl_2 under Ar. $PhSiH_2(OiPr)$ (15µL, 0.0858 mmol, 2 eq) and TBHP (5.5 M in decane, 16 µL, 0.0858 mmol, 2 eq) were added in sequence. The mixture was stirred at room temperature for 50 min. Saturated $Na_2S_2O_3$ were added. The layers were separated and the aqueous layer was extracted several times with EtOAc. The combined organic extracts were washed with brine, dried over $MgSO_4$, and concentrated. Purification by silica gel chromatography (hexanes:EA = 2:1) afforded the product as white solid 6.4 mg (yield 32%, brsm: 62%) and recovered nimbolide 9.6 mg (yield 48%).

[α] $_D^{25}$ = + 119.22 (c 0.253, MeOH), (literature**[α] $_D^{25}$** = + 122.2 (c 0.1, MeOH))

^{1}H NMR (600 MHz, $CDCl_3$): 7.33 (t, *J* = 1.7 Hz, 1H), 7.26 – 7.24 (m, 1H), 6.32 (dd, *J* = 1.9, 0.9 Hz, 1H), 5.53 (ddt, *J* = 8.4, 6.6, 1.9 Hz, 1H), 4.56 (dd, *J* = 12.1, 3.5 Hz, 1H), 4.21 (d, *J* = 3.5 Hz, 1H), 3.67 (dd, *J* = 8.5, 1.8 Hz, 1H), 3.56 (s, 3H), 2.86 (dd, *J* = 15.7, 5.2 Hz, 1H), 2.81 (ddd, *J* = 16.2, 11.6, 8.5 Hz, 1H), 2.71 – 2.67 (m, 2H), 2.40 – 2.35 (m, 1H), 2.32 (dd, *J* = 15.7, 5.8 Hz, 1H), 2.22 (dd, *J* = 12.1, 6.7 Hz, 1H), 2.14 – 2.08 (m, 3H), 1.70 (d, *J* = 1.8 Hz, 3H), 1.50 (s, 3H), 1.33 (s, 3H), 1.28 (s, 3H) ppm.

^{13}**C NMR (150 MHz, CDCl$_3$):** δ 210.42, 177.70, 172.90, 144.99, 143.01, 138.89, 135.97, 126.52, 110.38, 88.31, 82.77, 72.71, 51.64, 50.02, 49.60, 49.48, 49.33, 41.16, 40.79 (2C), 34.37, 33.22, 32.87, 17.06, 15.72, 15.09, 12.81 ppm.

HRMS (ESI-TOF): calc'd for $C_{27}H_{32}O_7$ $[M+H]^+$: 469.2216, found: 469.2221.

TLC: R_f = 0.4 (1:1 hexanes : ethyl acetate).

Compound 2

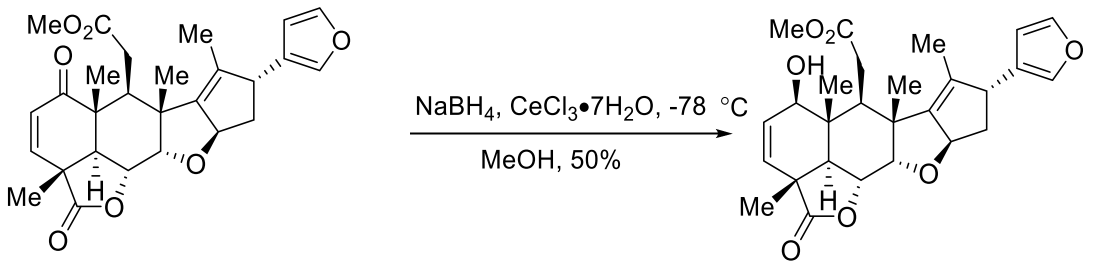

1

2

Nimbolide (25 mg, 0.054 mmol) was dissolved in 3 mL MeOH. The mixture was cooled to -78 °C and $CeCl_3 \cdot 7H_2O$ (40.3 mg, 0.108 mmol, 2 eq) was added followed by $NaBH_4$(4.9 mg, 0.108 mmol, 2 eq). After stirring at -78 °C for 30 min, the reaction was quenched by 30 mL saturated NH_4Cl then warmed to rt. Another 30 mL H_2O was added. The layers were separated and the aqueous layer was extracted with EtOAc (30 mL × 6). The combined organic extracts were washed with brine, dried over $MgSO_4$, and concentrated give the crude. Purification by silica gel chromatography (hexanes:EA 1:1.5) afforded the product as white solid 12 mg (yield 50%).

[α] $_{\mathbf{D}}^{\mathbf{26}}$ = + 38.01 (c 0.1, CHCl$_3$).

^{1}**H NMR (400 MHz, CDCl$_3$):** δ 7.34 (t, *J* = 1.7 Hz, 1H), 7.21 (s, 1H), 6.24 (d, *J* = 1.6 Hz, 1H), 6.16 (dd, *J* = 9.9, 2.4 Hz, 1H), 5.50 (dd, *J* = 9.9, 2.4 Hz, 1H), 5.48 – 5.41 (m,

1H), 4.51 (dd, J = 12.3, 3.7 Hz, 1H), 4.24 (dd, J = 8.0, 3.1 Hz, 2H), 3.67 (d, J = 8.6 Hz, 1H), 3.54 (s, 3H), 2.82 (dd, J = 15.5, 6.3 Hz, 1H), 2.56 (d, J = 12.3 Hz, 1H), 2.40 (dd, J = 15.5, 5.2 Hz, 1H), 2.30 (t, J = 5.7 Hz, 1H), 2.23 (dd, J = 12.1, 6.6 Hz, 1H), 2.17 – 2.10 (m, 1H), 1.74 (d, J = 1.9 Hz, 3H), 1.35 (s, 3H), 1.32 (s, 3H), 1.06 (s, 3H) ppm.

^{13}C NMR (100 MHz, $CDCl_3$): δ 176.74, 174.75, 145.54, 143.29, 138.95, 136.16, 133.14, 130.66, 126.84, 110.53, 88.33, 83.06, 74.48, 52.14, 50.29, 49.60, 47.55, 46.73, 43.61, 41.41, 40.75, 32.20, 29.85, 19.15, 16.64, 13.09, 12.81 ppm.

HRMS (ESI-TOF): calc'd for $C_{27}H_{32}O_7$ $[M+H]^+$: 469.2221, found: 469.2221.

TLC: R_f = 0.4 (EA:hexanes 1:1)

Compound 4

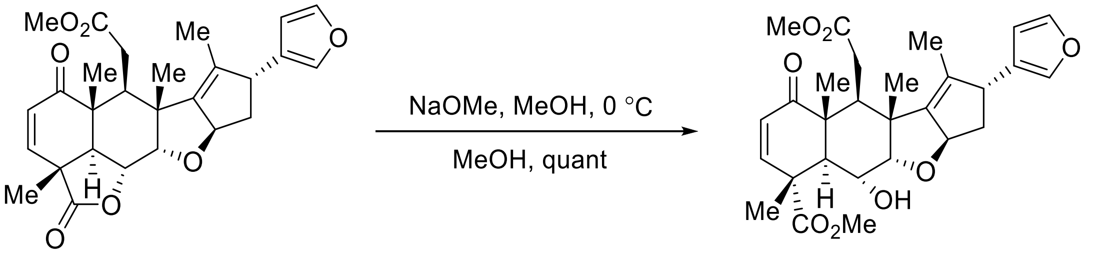

1

4

Under Ar (g), nimbolide (20.8 mg, 0.0446 mmol) and NaOMe (7.2 mg, 0.13 mmol, 3 eq) were dissolved in 3 mL MeOH at 0 °C. After stirring at 0 °C for 1 hour, the reaction was completed. Solvent was removed under vacuo, then the crude was purified on silica gel chromatography (hexanes:EA 1:1.5) to afford the product as white solid 22.1 mg (quant).

[α] **D**^{24} = +101.16 (c 0.17, $CHCl_3$) (**[α]** **D**^{20} = + 110 (c 1, $CHCl_3$)

^{1}H NMR (600 MHz, $CDCl_3$): δ 7.33 (t, *J* = 1.7 Hz, 1H), 7.24 (d, *J* = 1.1 Hz, 1H), 6.41 (d, *J* = 10.1 Hz, 1H), 6.33 (dd, *J* = 1.9, 0.9 Hz, 1H), 5.85 (d, *J* = 10.1 Hz, 1H), 5.55 (ddt, *J* = 8.4, 6.6, 2.0 Hz, 1H), 4.02 (d, *J* = 3.3 Hz, 1H), 3.92 (dd, *J* = 11.7, 3.3 Hz, 1H), 3.70 (s, 3H), 3.67 (s, 1H), 3.66 (s, 3H), 3.40 (d, *J* = 11.7 Hz, 1H), 2.90 (dd, *J* = 16.4, 5.7 Hz, 1H), 2.76 (dd, *J* = 5.7, 3.8 Hz, 1H), 2.26 – 2.20 (m, 1H), 2.20 – 2.17 (m, 1H), 2.04 (dt, *J* = 11.9, 8.5 Hz, 1H), 1.68 (d, *J* = 1.9 Hz, 3H), 1.59 (s, 3H), 1.29 (s, 3H), 1.21 (s, 3H) ppm.

^{13}C NMR (150 MHz, $CDCl_3$): δ 202.25, 175.61, 173.73, 148.14, 146.84, 143.12, 139.05, 134.98, 126.86, 126.47, 110.48, 87.44, 86.97, 66.25, 53.08, 51.73, 49.65, 47.79, 47.51, 47.37, 43.68, 41.48, 39.10, 34.42, 17.58, 17.19, 16.45, 12.91ppm.

HRMS (ESI-TOF): calc'd for $C_{28}H_{34}O_8$ $[M+H]^+$: 499.2319, found: 499.2326.

TLC: R_f = 0.4 (CH_2Cl_2:EA 6:1)

Compound 5

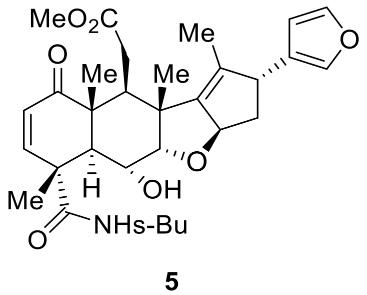

5

The compound 5 was prepared following reported procedure.

^{1}H NMR spectra of compound **3** in $CDCl_3$ (600 MHz)

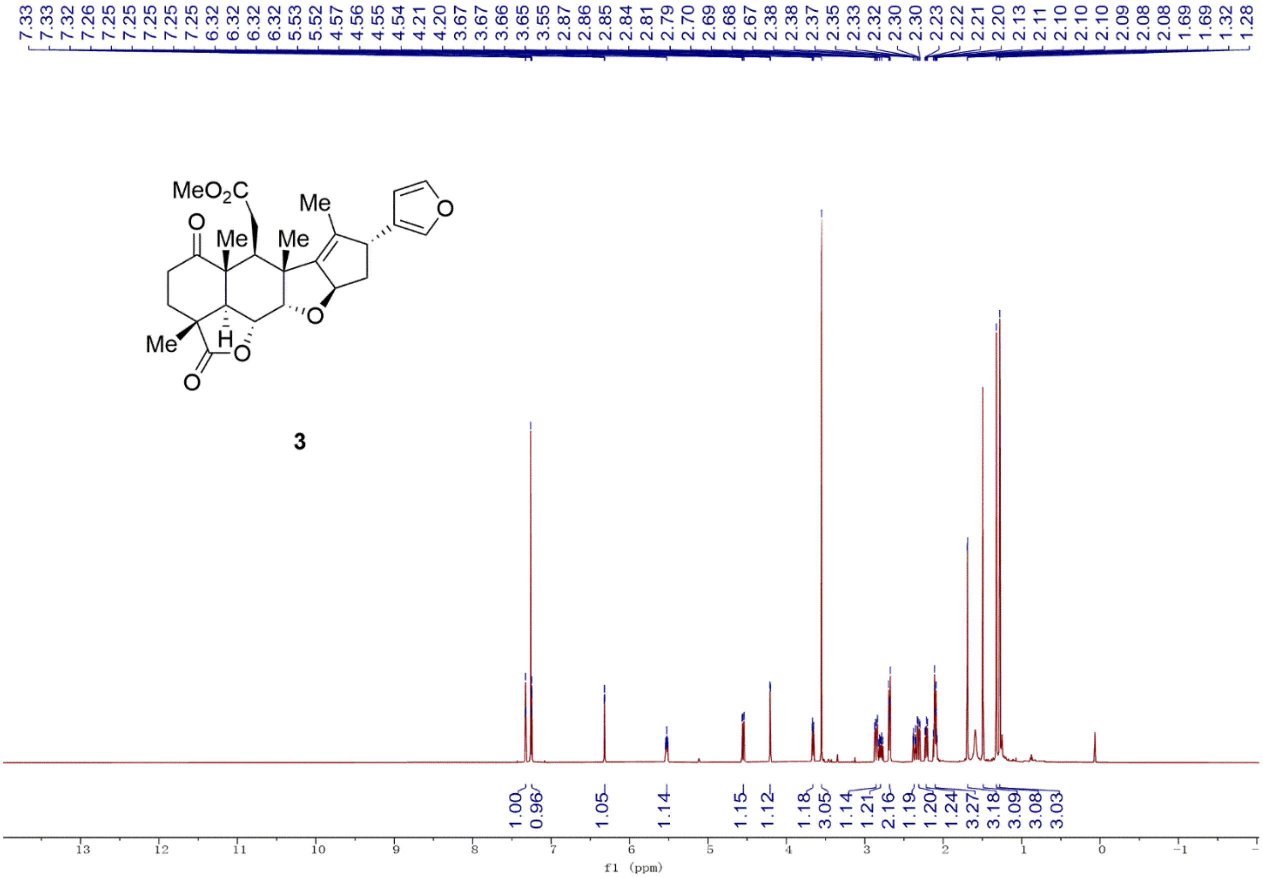

^{13}C NMR spectra of compound **3** in $CDCl_3$ (150 MHz)

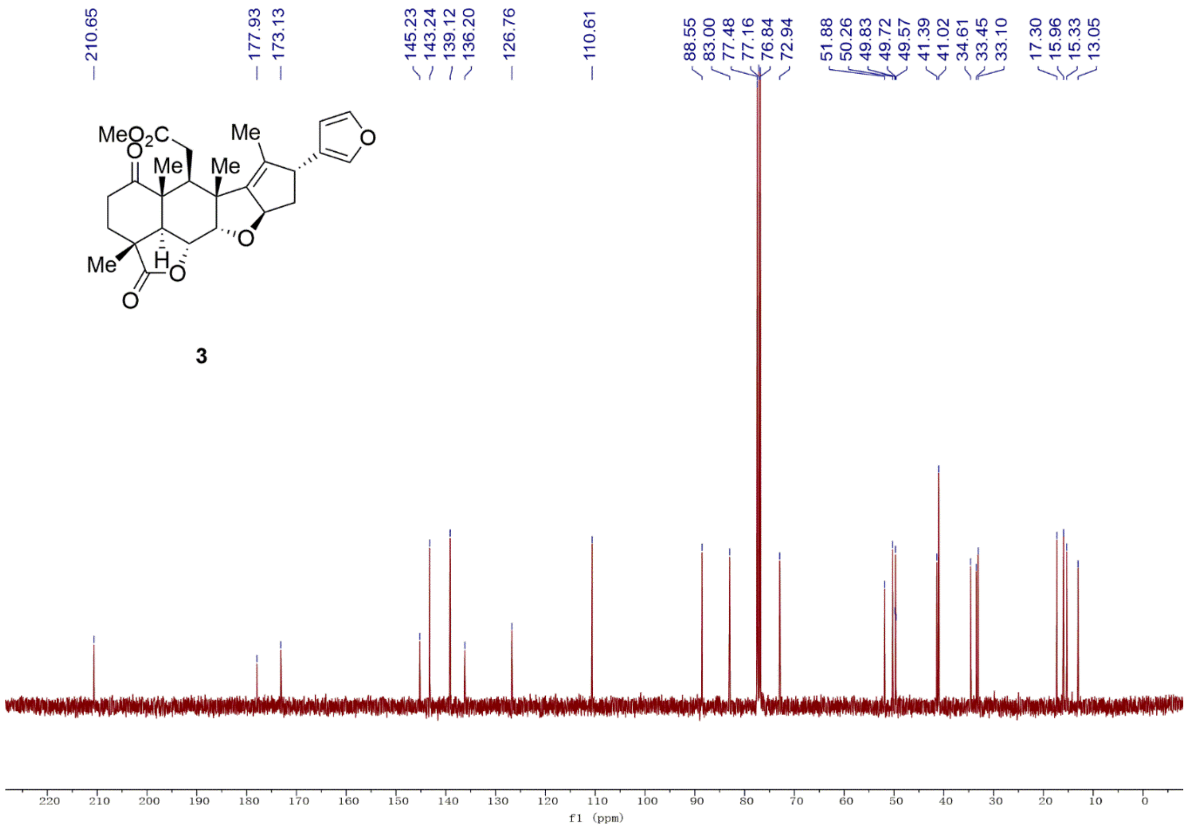

^{1}H NMR spectra of compound **2** in $CDCl_3$ (400 MHz)

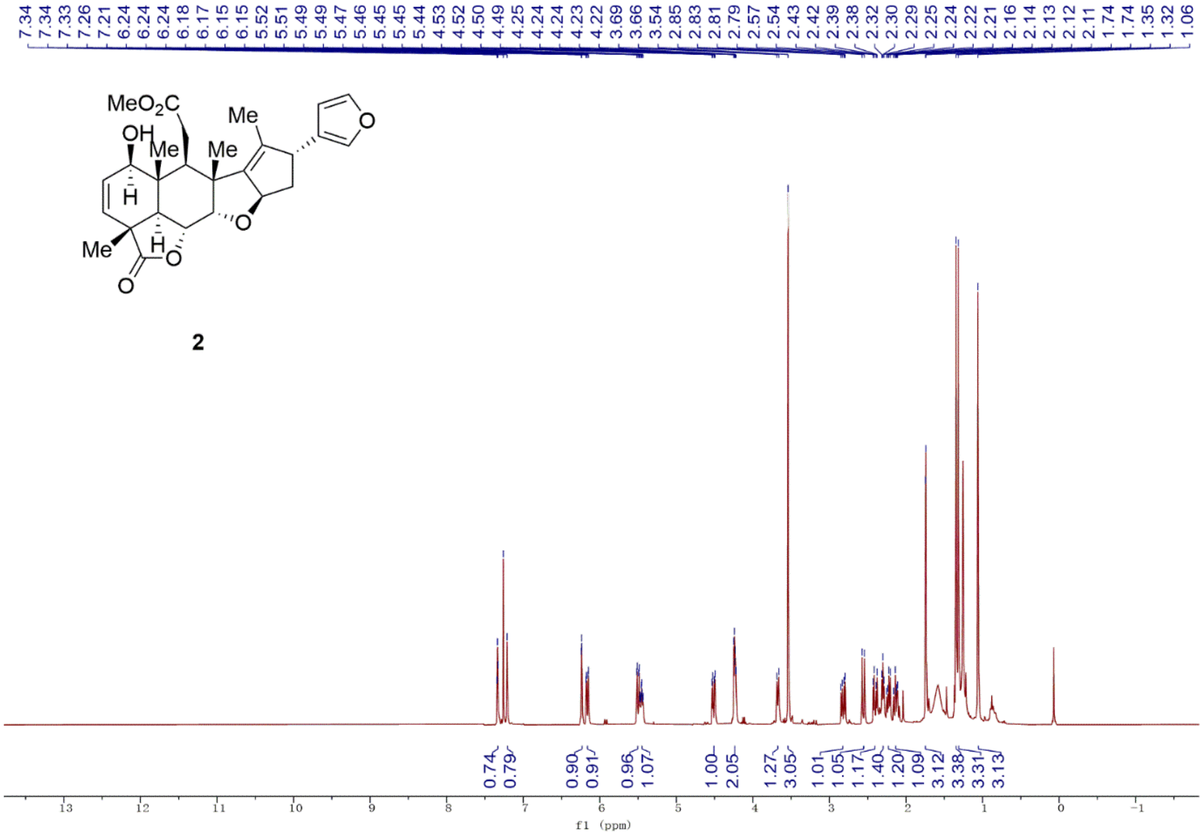

^{13}C NMR spectra of compound **2** in $CDCl_3$ (100 MHz)

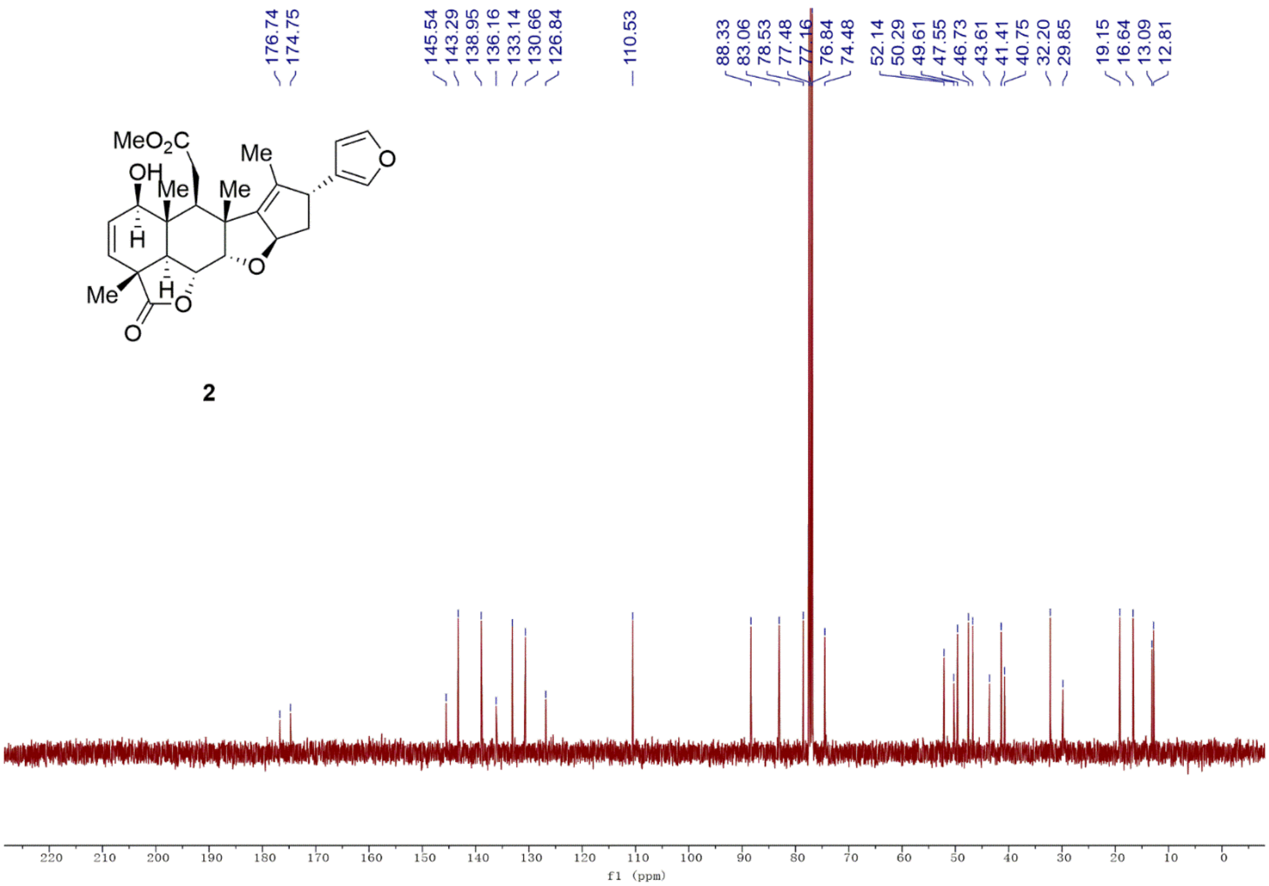

H-H NOESY of compound **2** in $CDCl_3$ (400 MHz)

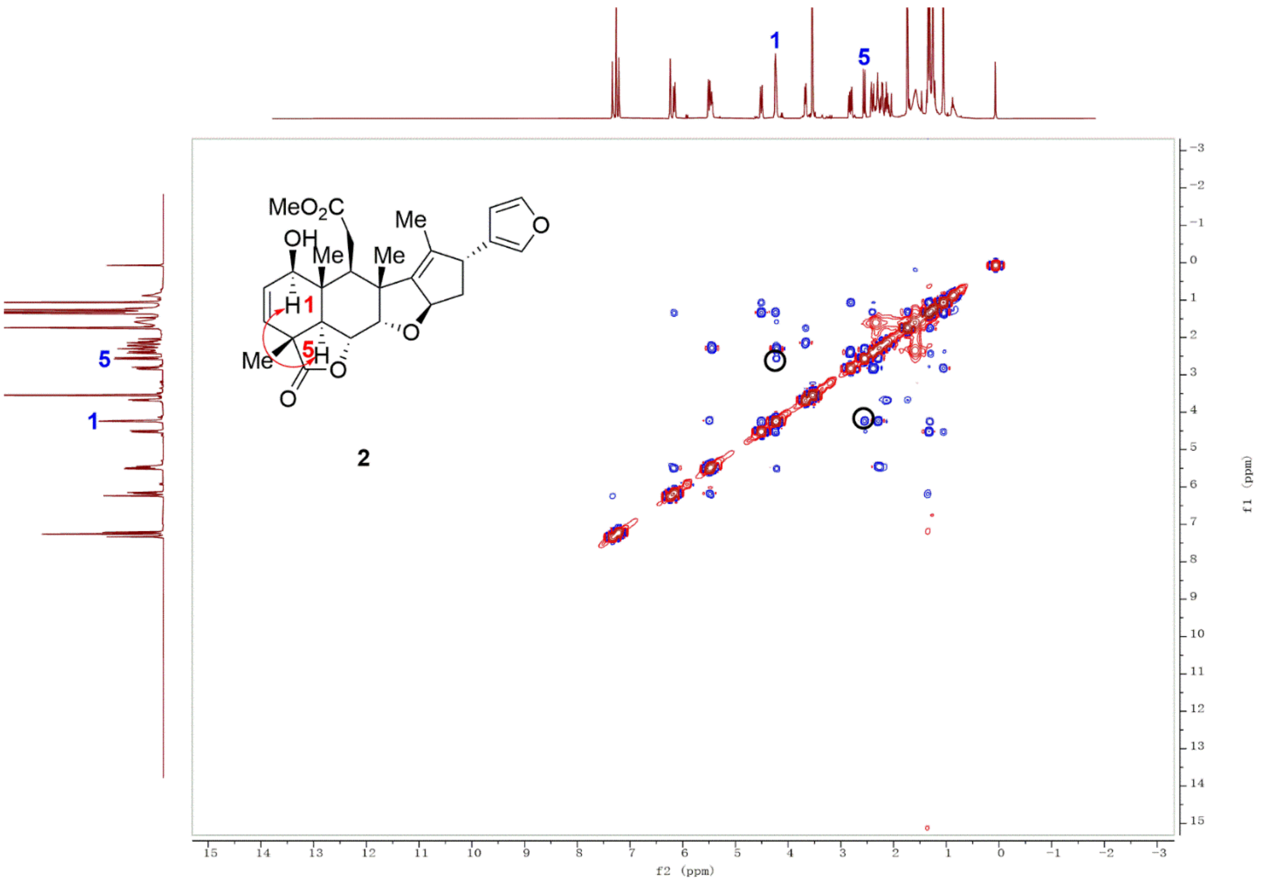

^{1}H NMR spectra of compound **4** in $CDCl_3$ (600 MHz)

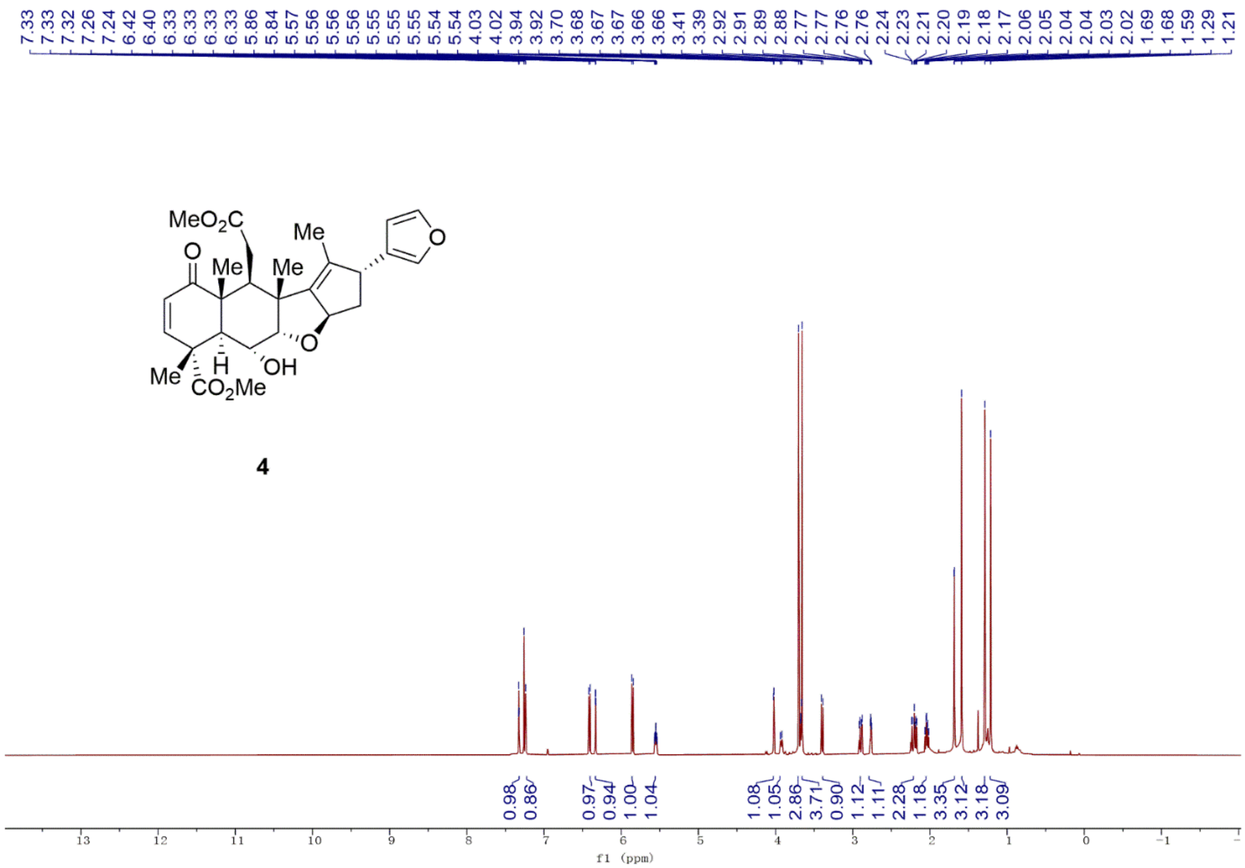

^{13}C NMR spectra of compound **4** in $CDCl_3$ (150 MHz)

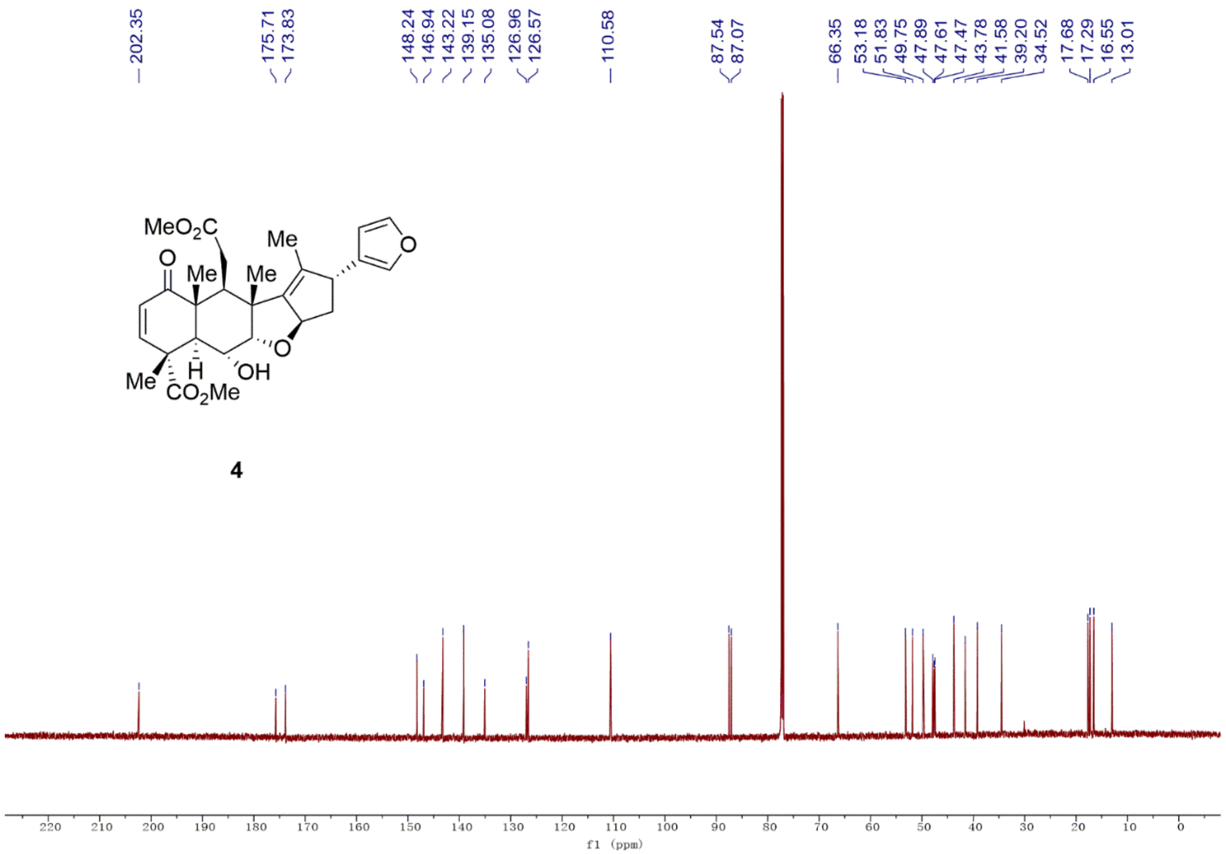
202.35
175.71
173.83
148.24
146.94
143.22
139.15
135.08
126.96
126.57
110.58
87.54
87.07
66.35
53.18
51.83
49.75
47.89
47.61
47.47
43.78
41.58
39.20
34.52
17.68
17.29
16.55
13.01
MeO2C
Me
O
Me
Me
O
H
OH
Me
CO2Me
O
4
220 210 200 190 180 170 160 150 140 130 120 110 100 90 80 70 60 50 40 30 20 10 0
f1 (ppm)

table S1. The quantitative proteomic MS experiments to identify the chromatin associate proteins.
